# Inductive sensing of magnetic microrobots under actuation by rotating magnetic fields

**DOI:** 10.1093/pnasnexus/pgad297

**Published:** 2023-09-12

**Authors:** Michael G Christiansen, Lucien R Stöcklin, Cameron Forbrigger, Shashaank Abhinav Venkatesh, Simone Schuerle

**Affiliations:** Department of Health Sciences and Technology, ETH Zurich, Zurich 8092, Switzerland; Department of Health Sciences and Technology, ETH Zurich, Zurich 8092, Switzerland; Department of Biosystems Science and Engineering, ETH Zurich, Basel 4058, Switzerland; Department of Health Sciences and Technology, ETH Zurich, Zurich 8092, Switzerland; Department of Health Sciences and Technology, ETH Zurich, Zurich 8092, Switzerland; Department of Biomedical Engineering, National University of Singapore, Singapore 117575, Singapore; Department of Health Sciences and Technology, ETH Zurich, Zurich 8092, Switzerland

**Keywords:** microrobots, inductive detection, magnetic control, rotational magnetic spectroscopy, magnetic particle imaging

## Abstract

The engineering space for magnetically manipulated biomedical microrobots is rapidly expanding. This includes synthetic, bioinspired, and biohybrid designs, some of which may eventually assume clinical roles aiding drug delivery or performing other therapeutic functions. Actuating these microrobots with rotating magnetic fields (RMFs) and the magnetic torques they exert offers the advantages of efficient mechanical energy transfer and scalable instrumentation. Nevertheless, closed-loop control still requires a complementary noninvasive imaging modality to reveal position and trajectory, such as ultrasound or X-rays, increasing complexity and posing a barrier to use. Here, we investigate the possibility of combining actuation and sensing via inductive detection of model microrobots under field magnitudes ranging from 100 s of microtesla to 10 s of millitesla rotating at 1 to 100 Hz. A prototype apparatus accomplishes this using adjustment mechanisms for both phase and amplitude to finely balance sense and compensation coils, suppressing the background signal of the driving RMF by 90 dB. Rather than relying on frequency decomposition to analyze signals, we show that, for rotational actuation, phase decomposition is more appropriate. We demonstrate inductive detection of a micromagnet placed in two distinct viscous environments using RMFs with fixed and time-varying frequencies. Finally, we show how magnetostatic selection fields can spatially isolate inductive signals from a micromagnet actuated by an RMF, with the resolution set by the relative magnitude of the selection field and the RMF. The concepts developed here lay a foundation for future closed-loop control schemes for magnetic microrobots based on simultaneous inductive sensing and actuation.

**Significance Statement**

Magnetic microrobots are anticipated to eventually be able to navigate the body and perform medical functions. While the use of magnetic fields to control their motion is well studied, getting real-time feedback on their position and movement usually requires a parallel imaging technique. Here, we use magnetic fields to both sense and actuate microrobots at the same time, designing a setup that uses symmetry to isolate voltages induced by the stray fields of model microrobots. We consider how inductive signal processing must differ from the more familiar approach used in magnetic particle imaging. Moreover, we demonstrate how inductive signals can be isolated to points in space under rotational actuation when the rotating field is combined with a selection field.

## Introduction

Magnetic stimuli offer an appealing means to control medical microrobots by providing noninvasive access to locations deep within the body (1–4). Limitations in scaling up magnetic field gradients to the dimensions of human patients, particularly gradients sufficient to manipulate the smallest microrobots, have fueled interest in locomotion schemes that are instead driven by magnetic torques (4–6). Microrobots have, for instance, been engineered to swim through fluids in a corkscrew motion (7, 8) or tumble (9–11) or roll (12–15) along surfaces in response to comparatively scalable magnetic fields with magnitudes of millitesla rotating at frequencies of Hz to 100 s of Hz. When applied to individual microrobots or swarms of microrobots (16), these rotating magnetic fields (RMFs) perform mechanical work through torque, transferring energy from the circuits driving the surrounding electromagnets to the microrobots and their surroundings (7, 8, 12, 16).

One of the longstanding challenges for the deployment of medical microrobots in relevant physiological situations like site-specific drug delivery is the implementation of closed-loop control, which typically requires simultaneous imaging and actuation (17, 18). While efforts have been made toward using complementary imaging techniques based on ultrasound or X-rays, magnetically based imaging modalities remain particularly appealing for this purpose because they avoid ionizing radiation and can potentially make dual use of the same apparatus for imaging and control (19). One approach has been to use magnetic resonance imaging systems in a rapidly multiplexed fashion for real-time imaging and control of microrobots with magnetic field gradients (20, 21). Nevertheless, the gradients required for guidance and imaging are likely to be different at any given instant (19), and using forces as a basis for microrobotic actuation has significant practical disadvantages compared with torques (5).

**Fig. 1.**
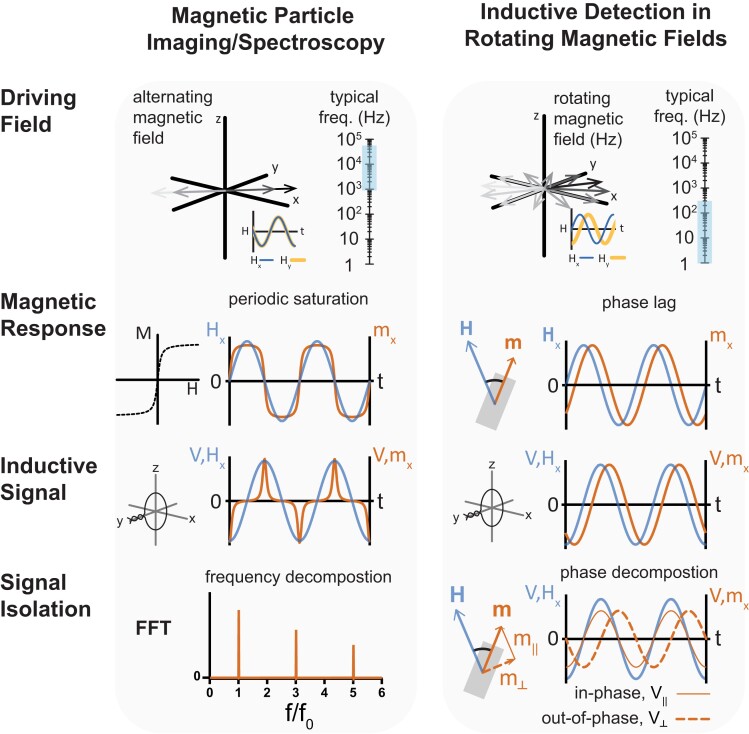
Graphical chart comparing salient conceptual aspects of signal acquisition and processing in the familiar case of MPI/spectroscopy with inductive detection of microrobots in RMFs. The driving field in the case of RMFs has a lower frequency and constant magnitude, leading to magnetic responses without periodic saturation. Consequently, the inductive signals expected from microrobots in RMFs generally do not contain higher order harmonics. Rather than decomposing their signals into different frequency contributions, it is more informative to decompose them in terms of different phase contributions at the same frequency. $H_{x}$ is the *x* component of the magnetic field *H*, $m_{x}$ is the *x* projection of the magnetic moment *m*, *f* is frequency, $f_{0}$ is the principal harmonic in the FFT, $V_{H_{x}}$ is the voltage induced by $H_{x}$, and $V_{m_{x}}$ is the voltage induced by $m_{x}$. Where applied as subscripts, ∥ and _┴_ denote in-phase or out-of-phase components, respectively.

A currently underexplored advantage of using RMFs for actuation is that the dynamic response of the microrobots themselves can generate inductively detectable voltage signals suitable for simultaneous feedback (22). This possibility for uninterrupted inductive detection during actuation is perhaps best appreciated through analogy to magnetic particle imaging (MPI), a technique in which voltage signals generated by the time-changing magnetization of magnetic nanomaterials are spatially selected with a selection field (SF) to reconstruct images (23, 24). Despite their similar underlying sensing principle, there are substantial and challenging technical differences between MPI and inductive detection with RMFs (Fig. 1). MPI employs much higher frequencies (kHz) with proportionally higher induced voltages, suggesting lower detection sensitivity for RMFs with a frequency below 100 Hz. Indeed, the magnetic stimuli required for MPI and RMF actuation are so distinct that it is possible to employ them sequentially on superparamagnetic swimming robots, albeit with lag during imaging sequences (25, 26). Here, we instead emphasize the possibility of inductively detecting the response of microrobots under the conditions used to actuate them. One important consequence of using RMFs rather than alternating magnetic fields to drive magnetization response is the elimination of the periodic saturation of magnetic material that leads to readily separable higher harmonic signal contributions (Fig. 1).

In this paper, we develop methodology and demonstrate a prototype for inductively detecting the response of individual microscale magnets to RMFs, which serve as model microrobots. The crucial task of canceling background signal from the RMF is accomplished through two separate mechanisms: a potentiometer that balances in-phase background signal and a set of orthogonal phase-adjusting loops alongside the compensation coil that can be selectively incorporated into the circuit to balance the out-of-phase background signal. Rather than relying on frequency decomposition to identify harmonic contributions from magnetic material as in MPI (24), we instead show that for inductive detection with RMFs, it is more relevant to consider phase decomposition. The out-of-phase component arising from the response of the microrobots is especially informative, in part because it is insensitive to unwanted diamagnetic or paramagnetic contributions, and because its magnitude can be related to irreversible work done on the microrobot(s). The expected relationship between design parameters of both the microrobotic component and the inductive detection apparatus is considered and elucidated. Finally, we show that inductive detection with RMFs is compatible with magnetostatic SFs, with the resolution of the zero point set by the relative field magnitude of the RMF and the selection field gradient. The techniques and concepts developed here represent a step toward the seamless integration of actuation and sensing of magnetic microrobots with scalable time-varying magnetic fields.

## Results and discussion

### Phase decomposition enables inductive detection with low-frequency RMFs

Like MPI or magnetic particle spectroscopy (MPS), inductive detection of microrobots actuated by RMFs involves the isolation of induced voltage signals produced by magnetically responsive materials surrounded by media with distinct and substantially weaker magnetic properties (24). In MPI and MPS, an alternating magnetic field is applied to drive periodic magnetic saturation, leading to readily separable higher order harmonic contributions to the inductively detected signal. In contrast, the use of an RMF, which remains constant in magnitude as it sweeps through a plane of rotation, precludes the possibility for signal isolation based on periodic saturation (Fig. 1). Instead, under the conditions of steady-state rotation that are often applicable to the locomotion of microrobots, the induced signal is purely sinusoidal, with a magnitude proportional to the moment of the microrobot and a phase shift attributable to the torque balance between viscous drag and magnetic torque (Fig. 1).

In the limit of zero drag, a microrobot rotates perfectly in-phase with an RMF, experiencing no instantaneous magnetic torque as it is carried along in its constant rotational velocity by its angular momentum. Under realistic conditions, the out-of-phase component of the magnetization corresponds to the magnetic torque being continuously applied to counteract drag or friction. While frictional and drag forces and torques are dissipative in the sense that they do irreversible work, magnetic microrobots actuated by RMFs are typically designed in such a way that the resulting convective flows or boundary interactions give rise to translational motion or surface walking (7, 9, 13). The out-of-phase component of the magnetization in these conditions is therefore useful to detect in part because its magnitude relates directly to the energy transfer from the field to the microrobot, a quantity that is often desirable to maximize. Another motivation for detecting the out-of-phase component of the magnetization is that this part of the signal has no analogous contribution from the paramagnetic or diamagnetic response of surrounding materials and thus might play a role similar to the higher frequency harmonics in MPI and MPS that arise uniquely from the behavior of the magnetic material (Fig. 1).

Phase decomposition of the detected signal from a microrobot can be expressed mathematically in several different ways. One computationally efficient method might be to perform a fast Fourier transform (FFT) (24) and consider the real and imaginary parts at the frequency of the RMF. Here, to facilitate comparison with the techniques we describe later for signals with time-varying frequencies, we choose instead to formulate the phase decomposition in terms of a Fourier integral with its basis limited to sines and cosines at the same frequency as the RMF. For a microrobot exposed to an RMF, the induced signal from the *x* projection of the magnetization, $V_{m_{x}}$, can be expected to vary as a function of time *t* as follows:


(1) $$V_{m_{x}}(t)=V_{∥}\sin\;\omega t+V_{⊥}\cos\;\omega t.$$

Here, *ω* is the angular frequency and $V_{∥}$ and $V_{⊥}$ are constant coefficients that can be determined through integration over one or more periods. Specifically,


(2) $$V_{∥}\;=\;\frac{\omega }{\pi }\int_{t_{i}}^{t_{f}}V_{m_{x}}(t)\sin\;(\omega t)dt,$$

where the limits of integration $t_{i}$ and $t_{f}$ are a single period apart. Analogously,


(3) $$V_{⊥}=\;\frac{\omega }{\pi }\int_{t_{i}}^{t_{f}}V_{m_{x}}(t)\cos\;(\omega t)dt.$$

Provided that (i) the signal induced by the *x* projection of the RMF, $V_{H_{x}}$, is in-phase with sin(ωt), and that (ii) the symmetry of the detection coil geometry ensures the true phase lag of the microrobot is undistorted in the detected signal, the coefficients $V_{∥}$ and $V_{⊥}$ describe the magnitude of the in-phase and out-of-phase contributions to $V_{m_{x}}(t)$, respectively (22). Due to the fundamental dependence of induced signals on the time derivative of magnetic flux density, the scale of voltage signals induced at different fixed frequencies are proportional to *ω*. This fact, taken with the coincidentally similar relationship between mechanical power transfer and frequency, implies that $V_{⊥}$ is directly proportional to the rate of irreversible work done on the microrobot. Alternatively, dividing $V_{∥}$ and $V_{⊥}$ by *ω* or frequency can enable comparison between normalized signals that are proportional to vector projections of the magnetic moment for measurements performed at different frequencies. These signals can be related back to the measured voltage by some factor of proportionality that depends on the geometry of the coils and amplification within the detection circuit, which could be determined using a sample with known properties similar to calibration of vibrating sample magnetometers (27). With this normalization, $V_{⊥}$ can be interpreted as proportional to the torque continuously applied by the field or, equivalently, proportional to the irreversible work done per cycle of the RMF.

### Background cancelation with rotating fields requires mechanisms for adjusting in-phase and out-of-phase contributions

While it would, in principle, be possible to use various alternative types of magnetic field sensors to detect stray field contributions from magnetic microrobots in an RMF, inductive detection is a favorable approach in practice because it permits designs for which the voltages induced by the RMF can be physically canceled within the coil itself prior to signal amplification. Often the measurement capabilities of such a setup are not limited directly by the scale of the detected signal voltage but rather by the extent to which the contribution of the microrobot is sufficiently distinguishable from whatever residual uncompensated signal remains from the driving RMF. While coil geometries that achieve perfect cancelation can be readily conceived abstractly, a suitable design for a real prototype must be combined with effective adjustment mechanisms that allow for fine-tuning of the background cancelation.

**Fig. 2.**
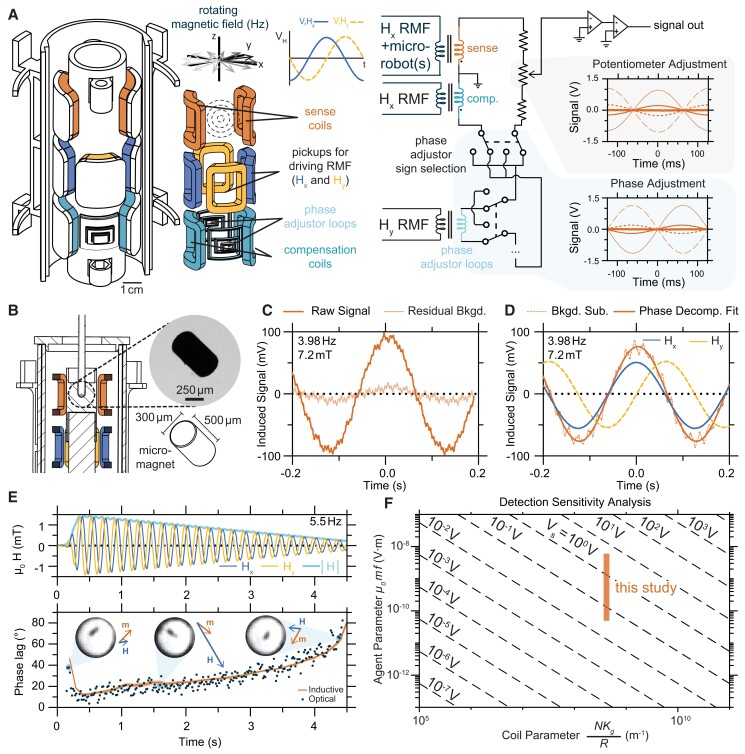
Inductive detection of a model microrobot actuated by a RMF. A) Sketch of prototype instrument for inductive detection, emphasizing the geometry of the pickup coils. (The coils supplying the RMF are omitted for clarity, but half of the support structure for the two-phase armature windings is depicted on the left.) Pickup coil pairs for sensing $H_{x}$ and $H_{y}$ reside at the center with 1,000 turns each, with the sense and compensation coil pairs with 5,000 turns each appearing above and below. Variably sized phase adjustment loops are included near the compensation coils. A simplified electrical schematic of the detection instrument is shown in the center right. For in-phase cancelation adjustment, the signal is taken from the wiper of a potentiometer. A plot of example signals generated by adjusting the potentiometer is provided at right. For out-of-phase cancelation adjustment, phase adjustment loops are selectively incorporated into the circuit with adjustable polarity using the partially represented array of double-pole-double-throw switches, with examples of their contributed out-of-phase signal shown on the bottom right. B) A cross-sectional detail of a sample holder situated within the detection apparatus. The model microrobot is a cylindrical micromagnet with a length of 500 µm and a diameter of 300 µm, suspended in water or pure glycerol. Brightfield micrograph of the micromagnet inset. C) Representative example of inductive signal collected for the microrobot in glycerol actuated by a magnetic field of 7.2 mT rotating at 3.98 Hz, with residual signal observed for an identical measurement on a vial containing only glycerol. D) The same signal is shown with the background subtracted, alongside the inductive signals measured by the field pickups. The fit is based on phase decomposition, as described in the text. E) At the top, a linear fade at a fixed frequency of 5.5 Hz was applied to a micromagnet simultaneously visualized at 60 frames per second to capture the ground truth of its response to the field. At the bottom, the instantaneous orientation determined by image analysis is compared with the inductively extracted phase lag between the field and the micromagnet. The connected curve is the mean of five technical replicates in which the sample was removed and replaced with a blank between each measurement, with the shaded bounds representing the 95% CI. F) A generalized parameter space predicting the scale of the measured induced voltage assuming a geometrically similar detection apparatus, with the range of predicted and observed values for this work highlighted by the indicated box. The abscissa is a parameter determined by the radius of the coil *R*, linear gain of the amplifier $K_{g}$, and number of turns *N*. The ordinate is a parameter set by characteristics of the microrobot, specifically its moment *m* and frequency of actuation *f*, as well as the permeability of free space $\mu_{0}$.

In the detection apparatus constructed for this work, the RMF was supplied by a two-phase set of armature coils arranged to generate an RMF within a cylindrical working volume in the plane perpendicular to the axis of the cylinder. At the center of the cylinder, on a 3D printed form, two sets of pickup coils were situated orthogonally to detect induced voltages from $H_{x}$ (blue) and $H_{y}$ (yellow) with a total of 1,000 turns each (Fig. 2A). These are required to monitor and record the time-varying components of fields applied to the working volume, for instance to ensure circular rotation and determine the phase shift between the RMF and the microrobot. Above these field pickups, a pair of coils with a total of 5,000 turns of 50 µm magnet wire was situated symmetrically on either side of the detection zone (orange, Fig. 2A). This set of coils is mirrored across the central plane of the cylinder by another set of coils with 5,000 turns that acts as the compensation coil (teal, Fig. 2A). These coils are connected in series as indicated in the schematic in Fig. 2A.

Background cancelation requires balancing the voltages between the sense coils and compensation coils as closely as possible in the absence of a microrobot. Because the driving field is an RMF, this means both the balance of their magnitude and their phase need to be finely adjusted (22). For in-phase amplitude adjustment, the signal was taken from the wiper of a 10-turn potentiometer before amplification, allowing for fine manual adjustment of the voltage divider (Fig. 2A, right inset). Multiple strategies can contribute to balancing the phase between these two coils. For rough cancelation, one strategy is to adjust the tension in the screws at top and bottom of the 3D printed coil holder to minutely adjust its precise angle with respect to the driving coil. For fine-tuning the phase cancelation in a systematic manner, a set of concentrically arranged orthogonal loops of varying areas (light blue, Fig. 2A) were formed and selectively incorporated into the sense and compensation coil circuit with double-pole-double-throw switches. The effective area of these loops varied by approximately a factor of 3.4 to 6 over four levels of adjustment, such that each set of five switches at the same level could span the out-of-phase signal contribution of a single switch at the next coarsest level (Fig. S1). This resulted in an array of 20 switches to provide adequate range and precision for the adjustment. The polarity of these loops could be changed with another switch (Fig. 2A), such that this scheme could introduce phase components either leading or lagging as needed. A typical adjustment involved iterative manipulation of the potentiometer and switches, incorporating the loops with the largest area first and using the smaller loops to attain progressively finer cancelation (Fig. 2A, right inset).

As a model microrobot, cylindrical neodymium iron boron micromagnets with 300 µm diameter and 500 µm length were employed in suspensions of water and pure glycerol (Fig. 2B). Despite their lack of immediate biomedical function, these micromagnets were adopted as a convenient model system that recapitulates the basic physics of rotating magnetic bodies in viscous media outlined in the previous section. The setup was compatible with a variety of sample holders employed throughout this study, tailored to the demands of each experiment, and a table fully summarizing their design and use is provided in the Supplementary material (Fig. S2). To observe signals from rotation of the micromagnets at the highest frequencies, the two-stage amplifier had to be reduced from an overall gain of approximately 120 to 70 dB (Fig. S3). A representative example of an averaged raw signal is shown in Fig. 2C, along with the residual background remaining when a vial without a micromagnet is placed back into the coil. A background subtracted signal is shown in Fig. 2D, alongside the voltage signals induced in the pickup coils for the field. The scale of this residual signal indicates that the cancelation scheme suppresses the induced signal from the driving field by 90 dB (Supplementary material text). By applying the mathematical techniques detailed in the previous sections, a fit was determined in terms of the coefficients $V_{∥}$ and $V_{⊥}$, and the resulting curve, which contains no contributions from 50 Hz noise, is shown as well.

To investigate how well the inductively sensed motion of a micromagnet corresponded to visual observations, a sample holder was employed in which the micromagnet could be simultaneously viewed from above by a camera. By exposing the micromagnet to a linearly faded RMF with a frequency of 5.5 Hz (Fig. 2E), it was possible to capture a progression toward step out behavior at a fixed frequency (Supplementary Movie S1). Using image analysis, the instantaneous orientation of the micromagnet was extracted and compared with the phase lag as determined by inductive signals (Fig. 2E). Because the initial orientation of the field is unknown in the videos, image analysis determines the “ground truth” phase lag only up to a constant offset that was adjusted to provide the best possible fit with the inductive data. The data from both methods agree well, but inductive detection offers the notable advantages of straightforward simultaneous measurement of the driving field and averaging during signal acquisition. The result is a more robust and less noisy determination of phase lag than image analysis alone could provide.

An additional validation test was performed by examining response of a micromagnet in glycerol and a micromagnet in water at a range of fixed frequencies spanning 1 to 100 Hz (Fig. S4). The results generally recapitulate the expected physical behavior for these systems. At low frequencies, both micromagnets rotate almost entirely in-phase with the field, with the out-of-phase component of the signal (and thus magnetic torque) increasing with frequency. The micromagnet in glycerol requires larger torques to maintain rotation at all frequencies, and its out-of-phase component reaches comparable values to the in-phase component at a lower frequency than the micromagnet in water. These observed behaviors comport well with analogous experiments performed using the well-established technique of rotational magnetic spectroscopy (28), which involves observing microscopic magnetic objects with brightfield microscopy as they rotate in RMFs of varying frequencies and magnitudes. The equations of motion in viscoelastic media have been described (29), including the regime of asynchronous motion above the critical frequency at which the micromagnet can no longer synchronously keep pace with the RMF. A somewhat analogous technique based on inductive detection called rotational drift spectroscopy also exists, which applies RMFs at 10 s of kHz to magnetic nanomaterials to observe shifts in hydrodynamic diameter in response to agglomeration or binding to analytes (30, 31). One key difference in the present work is the frequency range (1 to 100 Hz) set by the locomotion requirements for microrobots. This explains the need for coils with as many turns as possible and the emphasis on finding suitable mechanisms for finely tuned background cancelation.

Given the greatly reduced operational frequency required for simultaneous microrobotic actuation and detection, it is useful to consider the ultimate detection limits of this prototype and similar setups. A theoretical sensitivity analysis was performed by approximating the detection coils here as projected partial spherical surfaces (Fig. 2F; Supplementary material text and Fig. S5). The resulting scale of the voltage signals are plotted in terms of a quantity on one axis called the “agent parameter” that is determined by characteristics of the microrobot and a quantity on the other axis called the “coil parameter” that depends on aspects of the inductive detection apparatus. In terms of the how agent properties influence sensitivity, the detected voltage is proportional to the product of the magnetic moment of the microrobot and its preferred frequency of operation. If the properties of the microrobot are fixed and only aspects of the detection apparatus may vary, signals are proportional to the product of the number of turns and linear gain of the amplifier and inversely proportional to the linear dimensions of the coil geometry. The conditions explored experimentally in this work are mapped onto this space with the orange rectangle (Fig. 2F). Our work with this system suggests that it would be readily feasible to increase the gain of the amplifier and the number of turns for more sensitive detection (Fig. S3). We note, however, that this analysis does not account for other factors that may limit performance. These include the presence of a 50 Hz noise floor, thermal drift of the resistance balance between the sense and compensation coils, limits to the sensitivity of background cancelation, and the possibility for dielectric breakdown in the limit of coils with extreme numbers of turns that produce excessive internal voltages. These issues are all arguably addressable by design choices, and therefore, the scale of the sensed voltage represents a more fundamental constraint.

### Phase decomposition can be extended to rotating fields with time-varying frequencies

While using inductive detection to observe the response of a model microrobot to RMFs with discrete fixed frequencies is useful for capturing steady-state response, a more general and broadly applicable case is that of dynamic response to driving fields with time-varying frequencies. In a manner similar to variable frequency drives for industrial motors (32), an analogous technique can readily be envisioned for closed-loop control of microrobots with RMF frequency varied to optimize their inductively detected phase lag response. Here, the inductive detection apparatus would play a role equivalent to an analog resolver or other device that senses the phase lag of the rotor. To build toward real-time variable frequency drive optimization of microrobotic actuation, the phase decomposition technique shown in Eqs. 1–3 can be extended to the case of ω=ω(t). In such a case, $V_{∥}$ and $V_{⊥}$ can be treated as time-varying functions rather than constant coefficients, giving the following assumed functional form for $V_{m_{x}}(t)$:


(4) $$V_{m_{x}}(t)=V_{∥}(t)\sin\;[\omega (t)t]+V_{⊥}(t)\cos\;[\omega (t)t].$$

If $V_{∥}(t)$ and $V_{⊥}(t)$ vary slowly compared with the oscillation of the field and can be treated as approximately constant over one period, then it is possible to find approximate expressions for them at a time $t_{0}$.


(5) $$V_{∥}(t_{0})\approx \frac{2\int_{t_{i}}^{t_{f}}\;W(t)V_{m_{x}}(t)\sin\;[\omega (t)t]dt}{\omega (t_{f})t_{f}-\omega (t_{i})t_{i}}.$$

And


(6) $$V_{⊥}(t_{0})\approx \frac{2\int_{t_{i}}^{t_{f}}\;W(t)V_{m_{x}}(t)\cos\;[\omega (t)t]dt}{\omega (t_{f})t_{f}-\omega (t_{i})t_{i}}.$$

Here, W(t) is a weighting function, defined in terms of ω(t).


(7) $$W(t)\equiv \frac{d}{dt}[\omega (t)t].$$

The limits of integration must account for the shifting frequency to describe a single period, such that $t_{i}$ and $t_{f}$ are functionally related in terms of ω(t).


(8) $$\omega (t_{f})t_{f}=\omega (t_{i})t_{i}+2\pi .$$

The midpoint of $t_{i}$ and $t_{f}$ can be taken as the time $t_{0}$ corresponding to the approximate instantaneous value of the coefficient functions.

**Fig. 3.**
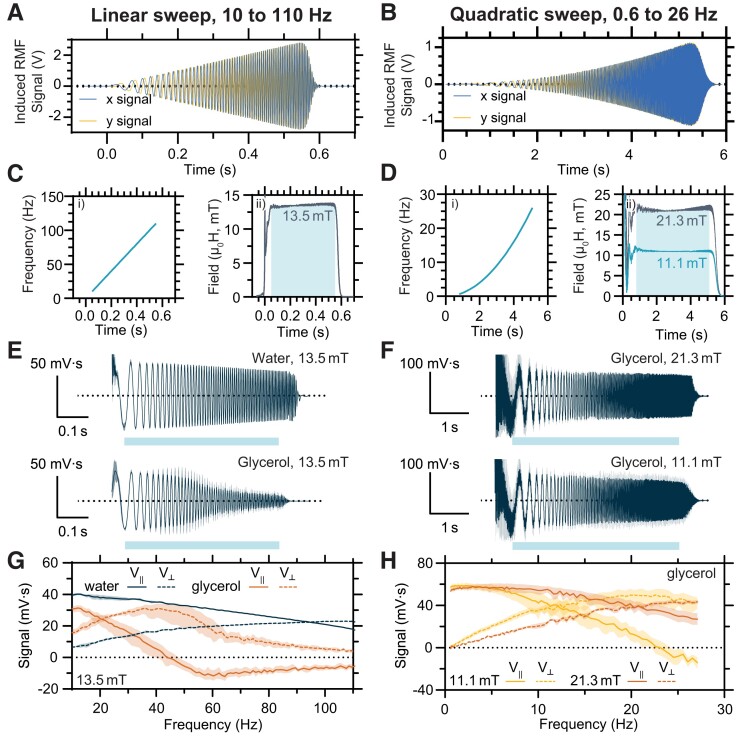
Phase decomposition for signals with time-varying frequencies. The raw inductive signals generated by the RMF, as detected by the $H_{x}$ and $H_{y}$ pickup coils for a rapid linear frequency sweep with constant field magnitude from approximately 10 to 110 Hz A) and a slow quadratic frequency sweep with constant field magnitude from approximately 0.6 to 26 Hz B). Frequency versus time as defined for the input waveforms (i) and field magnitude as inferred from the measured inductive signals as a function of time (ii) are shown for the linear sweep C) and the quadratic sweep D). The timeframe selected for phase decomposition analysis is indicated by the shaded region. Frequency normalized, background-subtracted signals are plotted for the same model microrobot as the previous figure under the annotated conditions for the linear sweep E) and quadratic sweep F). The dark curve is the mean of five technical replicates in which the sample removed and replaced with a blank between each measurement, with the shaded bounds representing the 95% CI. The light box below each plot represents the timeframe selected for decomposition analysis. The signal is decomposed into its instantaneous in-phase component $V_{∥}$ and out-of-phase component $V_{⊥}$ and plotted as a function of frequency for the linear sweep in G) and quadratic sweep in H). The linear sweep is used to probe the response of nominally identical micromagnets in the distinct viscous environments of water and glycerol. The quadratic sweep is used to probe the variation in the response of a single micromagnet to different driving field amplitudes. Curves represent the mean of five technical replicates in which the sample removed and replaced with a blank between each measurement, with shaded regions representing the 95% CI.

To investigate the possibility of processing signals in this manner, we exposed the same micromagnets from the previous section to RMFs of constant magnitude with time-varying frequencies. Specifically, we made use of a rapid linear frequency sweep from 10 to 110 Hz (Fig. 3A) and a slower quadratic frequency sweep from approximately 0.6 to 26 Hz (Fig. 3B). For the linear sweep, the driving field advanced through the full frequency range in less than a second while its amplitude was kept constant (Fig. 3C). The relatively wide frequency range of the linear sweep allowed the effect of the same driving conditions to be tested on nominally identical micromagnets in two different viscous environments, glycerol and water. At 20°C, the viscosity of glycerol is 1,420 mPa s, more than 10^3^ times greater than that expected for water, 1.005 mPa s (33). The quadratic sweep was chosen to show that the methods developed here are also applicable to nonlinear frequency-versus-time functions. In theory, any frequency function could be chosen, provided the assumption of slowly varying $V_{∥}$ and $V_{⊥}$ is reasonable. For the quadratic sweep, the frequency increased quadratically over 5 s, and the sweep was performed at two distinct amplitudes for the same microrobot in glycerol (Fig. 3D). Frequency normalized, background subtracted curves from inductive detection of the microrobot are shown in Fig. 3E and F. The magnitude of the signal can be observed to drop off rapidly with increasing frequency in the case of the micromagnet in glycerol under a linear sweep, and the signal persists after the magnitude of the field has fallen in the case of the micromagnet in water. Otherwise, by inspection, many of these frequency normalized curves appear similar, and phase decomposition analysis is needed to extract additional information.

Phase decompositions of the inductively detected signals from the linear sweep are shown in Fig. 3G and for the quadratic sweep in Fig. 3H, with instantaneous values for $V_{∥}(t)$ and $V_{⊥}(t)$ shown instead as a function of frequency, $V_{∥}(f)$ and $V_{⊥}(f)$, based on the known time dependence of the frequency. Further details about the signal processing procedure can be found in the text of the Supplementary Material and Fig. S6. In the case of microrobots under conditions of different viscosity, phase decomposition reveals response curves that appear to be functionally similar but shifted to lower frequencies for the microrobot in glycerol due to the substantially greater viscous drag. If the goal were to maximize $V_{⊥}$ to optimize mechanical energy transfer based on the environment of the microrobot, a much higher frequency would obviously be needed in water than in glycerol. Curves like these could offer a basis for selecting or testing the optimality of drive frequency in real time as a microrobot encounters differing local conditions. Similarly, Fig. 3H shows how phase decomposition reveals changes in frequency-dependent response as a function of the magnitude of the RMF, which determines the corresponding scale of available magnetic torque. Notably, the cross-over frequency between the in-phase and out-of-phase component of the signal can be seen to increase approximately proportionally to the RMF magnitude, as expected. At 11.1 mT, the cross-over frequency was 12.2 Hz, and at 20.7 mT, the cross-over frequency was 20.7 Hz. Wide or rapid frequency sweeps like the ones shown are most useful in the context of characterization, and for the purpose of closed-loop control, the frequency would likely be varied less rapidly and over a smaller range of values.

### Magnetostatic SFs allow for spatially restricted rotational actuation and inductive detection

Previous work has indicated the possibility of using a magnetostatic SF to spatially isolate the delivery of magnetic torque to individual microrobots or magnetic torque density to diffuse assemblies of microrobots or magnetic material (22, 34–38). Mechanistically, by combining RMFs and magnetostatic SFs, the rotational character of the field is preserved only in the field-free region and suppressed at points away from it where the magnitude of the superimposed SF becomes comparable to or higher than the RMF (34, 35). As discussed in a previous section, the out-of-phase component of the inductive signal detected from microrobots in an RMF reflects the mechanical work being done by magnetic torque. Together, these facts led us to hypothesize that inductive signals produced by microrobots in response to RMFs should also be spatially isolated by a magnetostatic SF, in a manner analogous to MPI. This would imply that, in addition to the possibilities for spatially selective rotational actuation that SFs offer, the inductive signals produced in them might also reveal positional information about the actuated microrobots.

**Fig. 4.**
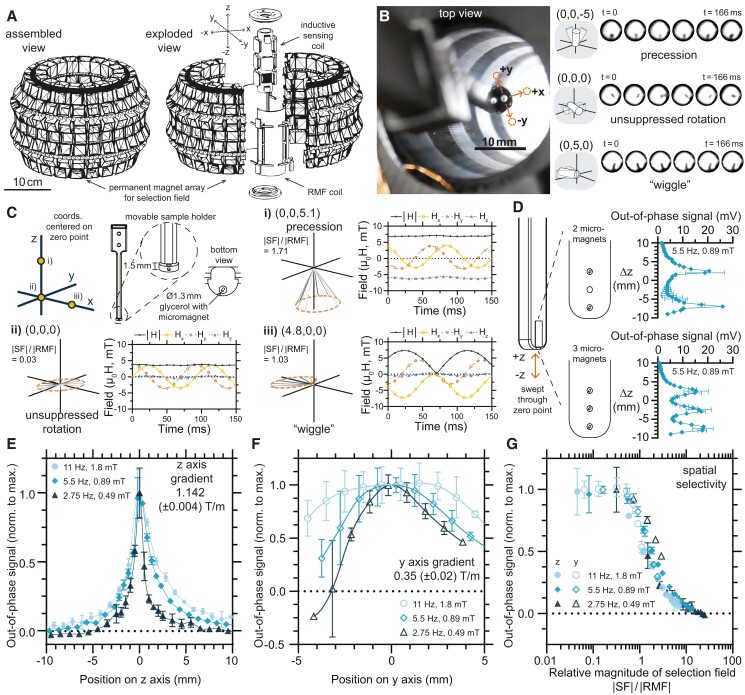
Magnetostatic SFs can spatially isolate torque delivery and inductive signal generation by micromagnets to field-free regions when combined with RMFs. A) Illustration of the array of permanent magnets used to surround the inductive detection apparatus and create a zero-field point within the inductively sensed region. B) A photograph from above shows a micromagnet in a chamber at the end of a positioner arm affixed to a micropositioner that moves sample precisely in the selection field. Cropped still frames from Movies S2 and S3 illustrating examples of each of the main types of motion observed: suppressed precession at points along the *z* axis, rotation near the zero point, and suppressed “wiggle” motion at points along the *x* or *y* axes. Also see Movies S2 and S3. C) Sketch of the sample chamber used to test inductive signal generation during visualization within a magnetostatic SF as a function of position, consisting cylindrical cavity filled with glycerol bearing a model cylindrical micromagnet (500 µm length, 300 µm diameter). Results of characterization of the net field produced by the combined SF and RMF are shown at several representative points: i) displaced along the positive *z* direction, ii) at the zero point, and iii) displaced along the positive *x* direction. Curves are sinusoidal fits of data taken with a Hall probe. D) An alternative holder containing two or three micromagnets was swept along the *z* axis through the zero point during actuation at 5.5 Hz and 0.89 mT, showing that the SF can be used to independently actuate and isolate signal from each micromagnet. Error bars are the SD of three technical replicates. E) and F) examine spatial resolution of the SF, showing the variation of the out-of-phase component of the inductive signal produced by the model micromagnet as a function of position as it is swept along the *z* axis (1.142 T/m) and *y* axis (0.35 T/m), respectively. Each curve represents measurements under distinct RMF conditions (1.84 mT at 11 Hz, 0.89 mT at 5.5 Hz, and 0.49 mT at 2.75 Hz) and is normalized to the maximum mean out-of-phase signal observed or interpolated by a second-order spline curve. Error bars represent 95% CI, with three technical replicates in which the sample arm was detached and reattached between each series. G) Relative magnitude of the SF, defined as the quotient of the SF magnitude experienced at a point to the magnitude of the superimposed RMF, is used to replot the normalized out-of-phase signal data from positive displacements in E) and F).

To test this hypothesis, we constructed an array of permanent magnets capable of fully enclosing the inductive detection setup described in previous sections, which established a SF with a zero point that resided within the working volume of the inductive sensing apparatus (Fig. 4A; Fig. S7). This array was based on a “magic sphere” geometry—a modified azimuthal revolution of a second-order Halbach cylinder with a central field-free point (39, 40). In this case, as with a similar array that we designed previously for torque density focusing (34), a 3D printed support structure held ferrite ceramic permanent magnets (grade Y35) that were stacked to form uniformly magnetized cubes with an approximately 2 cm side length. On the inside of the partial sphere, around its equator, neodymium iron boron magnets were interleaved with the ferrite magnets to improve the resolution of the SF along the *x* and *y* axes. The results of a finite element model predicting the magnetic field generated by an axisymmetric approximation of this configuration of permanent magnets is shown in Fig. S8. This simulation is best interpreted as an upper bound for the expected field under the assumption of a perfect circumferential packing factor. This geometry is expected to produce its strongest gradient in the *z* direction, and indeed the largest gradient observed (1.1 T/m) was measured along the *z* axis using a Hall probe controlled by a micropositioner (Fig. 4C; Figs. S7 and S8). The gradients observed along the *x* axis (0.8 T/m) and along the *y* axis (0.4 T/m) were unequal due to a column of omitted magnets along each side of the seam where the two halves of the sphere met. Although an isotropic zero point would likely be desirable for imaging or inferring position, in this case, the ellipsoidal field-free region was useful because it permitted measurements of inductive signal at known positions along different magnetostatic gradients without reconfiguring the setup (Fig. S8).

To confine a model microrobot to a precisely movable location, a micromagnet nominally identical to the ones employed in the previous sections was placed in a 3D printed 1.3-mm-diameter cylindrical cavity filled with glycerol and sealed on either side with a polycarbonate sheet to allow visualization from above (Fig. 4B). The sample chamber was affixed to a 3D printed polymer arm mounted on a micropositioner so that it could be moved throughout the working space (Figs. S2 and S7). While it is possible to electronically displace the position of a field-free region using additional coils to supply an offset field, in the experiments that follow, we instead elected to move sample holders within the working volume, an approach that is equivalent from the perspective of the micromagnet. As expected, in the immediate vicinity of the zero point, the magnet exhibited unsuppressed rotation (Fig. 4B). At points along the *z* axis, the out-of-phase signal also drops, and the micromagnet exhibits precession (Fig. 4B; Fig. S9 and Supplementary Movie S2). At points away from the zero point along the *x* or *y* axis, the micromagnet “wiggles” rather than fully rotating and the out-of-phase inductive signal drops (Fig. 4B; Fig. S9 and Supplementary Movie S3). The basis for these observed behaviors becomes clear when the net field resulting from the superposition of the SF and the RMF at these points is measured with a Hall probe, with several representative examples shown in Fig. 4C. For points along the *z* axis displaced from the center, the net field is similarly observed to undergo precession about the *z* axis (Fig. 4C, i). At the zero point, the field is measured to rotate in the *xy* plane with a magnetostatic contribution less than 0.1 mT (Fig. 4C, ii). At points along the *x* or *y* axis, the magnetostatic contribution occurs entirely within the *xy* plane, leading to “wiggling” and suppressing the rotational character of the field (Fig. 4C, iii). Away from the principal axes, behavior intermediate between these special cases can be expected.

Given that a combined SF and an RMF can isolate torque delivery to a single point, as in the described apparatus, it ought to be possible to independently actuate micromagnets that are confined to separate locations. In the context of inductive detection, this selective delivery of torque is associated with an observable out-of-phase voltage signal. To investigate whether torque could be selectively delivered to multiple micromagnets, a sample holder was designed in which two or three micromagnets were confined to spherical cavities filled with glycerol and separated by 10 or 5 mm, respectively (Fig. 4D). The holder was aligned with the zero point and moved along the *z* axis so that each of the sample chambers was swept through the zero point during exposure to a continuous RMF at 5.5 Hz. The result, shown in Fig. 4D, is that peaks associated with delivered torque exhibit the same number and spacing as the micromagnets loaded into the sample holder, with the more closely spaced micromagnets experiencing greater cross-talk in torque delivery.

To elucidate the influence of RMF and SF parameters on the resolution of the delivered torque, a sample holder with a single micromagnet in a spherical cavity filled with glycerol was moved by the micropositioner through a set of points along the *z* axis and *y* axis. The effective resolution of the zero point was anticipated to depend on RMF magnitude, with spatial selectivity increasing as RMF magnitude decreases (34). For the frequencies selected for this experiment, suitable RMF magnitudes were determined empirically to maximize the out-of-phase component of the inductively detected signal when the sample chamber was placed at the zero point—1.8 mT at 11 Hz, 0.89 mT at 5.5 Hz, and 0.49 mT at 2.75 Hz (Fig. S10). As expected, the high gradient of the SF along the *z* axis resulted in the highest observed spatial selectivity, with the out-of-phase inductive signal from the microrobot dropping to half its peak value within millimeters of the zero point (Fig. 4E). Along the weakest gradient of the zero point, corresponding to the *y* axis, spatial selection was also observed, though it was weaker (Fig. 4F). For the highest RMF magnitudes tested, a plateau is observed in the immediate vicinity of the zero point along the *y* axis, a behavior that vanishes as the RMF magnitude is decreased. To rule out the role of position-dependent inductive coupling to the sense coil as the origin of the spatial variation of these signals, the response of a magnet in glycerol was tested as a function of position in the absence of the SF (Fig. S11). Although the inductive signal varies measurably with position, as expected, the spatial dependence is far weaker than in the presence of the SF.

If the normalized out-of-phase signal data points from the positive displacements of Fig. 4E and F are replotted as a function of the relative magnitude of the SF to the RMF, the general behavior appears to converge toward a single curve (Fig. 4G). This is consistent with our recent finding that the spatial resolution achieved when combining a RMF and a magnetostatic SF is set primarily by the parameters of these two fields (34), a result that is intuitive in the context of vector superposition (Fig. 4C). The fact that resolution is set primarily by field parameters is an important difference from MPI, where material properties play a key role in setting spatial resolution (24).

## Conclusion and outlook

In this work, we have designed prototype instrumentation and demonstrated signal processing for simultaneous inductive detection and actuation of a model microrobot with low-frequency RMFs. Moreover, we showed that this form of inductive detection can be combined with a magnetostatic SF for restricting actuation and the inductive signal it generates to a single point. Our findings suggest that inductive detection of microrobots could serve as a basis for closed-loop control, whether directed toward optimizing mechanical energy transfer or controlling microrobot motion.

While actuating microrobots with time-varying magnetic fields strongly suggests an opportunity to detect their response continuously and simultaneously via induced voltage signals, it is also worthwhile to consider how this approach could compare with alternative methods for detecting magnetized microscale objects. The stray fields of microrobots could, in principle, instead be observed by magnetic field sensors, following similar methods to those demonstrated for capsule endoscopy (41, 42) or magnetomicrometry with millimeter-scale magnetic beads (43). In the present study, the field from the model microrobot experienced at the distance of the sensing loops is only about 1 µT, compared with the superimposed RMF on the order of 10 mT. This implies that Hall probes or other magnetic sensors would need to be able to detect a net field with a combined magnitude of 10 s of mT with at least ±0.01% precision at sample rates exceeding 10 s or 100 s of Hz in order to produce comparable data during simultaneous actuation. While perhaps possible, this would require a magnetic field sensor with an exceptionally high sensitivity and dynamic range. In contrast, in the work presented here, this isolation is accomplished primarily through geometric symmetry, requiring only simple components, inexpensive signal amplifier chips, and a robust strategy for fine-tuning cancelation of the signal from the driving field. Another substantial difference is in scaling behavior. We showed that by assuming the properties of the microrobot remain constant, and uniformly scaling up inductive sensing coils, signal decreases as 1/R (Fig. 2F). The scaling situation is substantially more restrictive for point measurements such as those provided by Hall sensors, which would simply drop off as $1/R^{3}$ as the distance from a source increases.

For the technical potential of inductive detection of microrobots to be more fully realized, further development is required. For one, similar techniques should be adapted for application to smaller microrobots such as bacteria-inspired helical swimmers (6), magnetically responsive bacteria (22), or surface microrollers (14). The micromagnet picked here as a model microrobot does adequately reflect the basic physics of these systems, but even smaller microrobots are more likely to be deployed in a medical context. Our sensitivity analysis indicated that induced voltage signals are inversely proportional to linear dimensions of pickup coils, so detecting smaller microrobots in realistic working volumes will require increasing the number of turns, boosting amplification, and shielding setups from ambient 50 Hz noise. The cancelation techniques that were demonstrated in this work can also be further refined, especially through automation, since here they were implemented manually. Ultimately, the form factors of greatest interest for real applications could be single sided (44, 45), i.e. based on detection coils that do not enclose the working volume, and executing this in practice would require studying how pickup coil geometry influences phase and magnitude of the induced signal. In this respect, the spatial isolation provided by the SF may also offer a practical path, since an integrated array of permanent magnets in a single-sided design could isolate inductive signal generation to a point or line with known inductive coupling behavior.

Several different forms of noninvasive energy transfer and actuation are available to control medical microrobots, including ultrasound, light, and magnetic fields. Each of these has its own unique features and drawbacks. Up to this point, one of the major limitations of magnetic microrobot control has been the need for complementary imaging, which remains an outstanding challenge. The realization of simultaneous magnetic actuation and inductive detection of microrobots with time-varying magnetic fields could represent a step toward feasible real-time closed-loop control.

## Materials and methods

### Construction of the inductive sensing setup

Custom-designed coil holders were 3D printed with a resin-based digital light processing printer (Anycubic Photon Mono X) and assembled with epoxy and plastic hardware. STL files have been made available in a data depository (46). Sense and compensation coils were wound by hand using enameled magnet wire with 50 µm diameter, a 100-µm-diameter magnet wire was used for the *x* and *y* field sensing coils, and a 200-µm-diameter magnet wire was used to wind the phase adjustment loops. The RMF coils were also wound by hand with 300 turns of magnet wire of 0.9 mm diameter, symmetrically alternating between the phases in groups of 10 turns to maintain overall symmetry. Signals were generated with a two-channel arbitrary function generator (Keysight EDU33212A) and fed to a two-channel class AB power amplifier with a rated maximum output power of 80 W per channel (Crown D-150A). The power amplifier was situated across the room from the setup to reduce 50 Hz noise. Pickup coils for sensing $H_{x}$ and $H_{y}$ were measured directly with an oscilloscope (Keysight DSOX2004A). The output from the sense and compensation coil pair was wired in series with the phase loop switchbox, which had one switch to determine sign of the out-of-phase contribution and could selectively incorporate up to 20 separate loops with four different magnitudes. Finally, the signal was taken from the wiper of a potentiometer and underwent two-stage signal amplification. Additional information on the amplifier design is provided in Fig. S1.

### Phase decomposition at fixed frequencies

Input signals were tuned manually to provide a constant target RMF magnitude and an appropriate quarter period phase shift between the $V_{H_{x}}$ and $V_{H_{y}}$ signals. The factor relating the induced voltage from the field probes to the RMF magnitude was found through empirical calibration at a single frequency via simultaneous measurements with a three-axis Hall probe (MetroLab THM1176). The micromagnet (SM Magnetics, Cyl0003-50) was placed in pure water or pure glycerol in a standard 5-mm NMR tube, and blank samples containing only glycerol or water were similarly prepared. At each frequency, five separate measurements of the voltage signal were performed, with blank measurements performed before and after each trial and internal averaging on the oscilloscope set to 64. Background cancelation was fine-tuned prior to each sequence of measurements but left untouched between measurements of blanks and trials. Mathematica was used to analyze the resulting data. In brief, for fixed frequency measurements, a sinusoidal function was fitted to $V_{H_{x}}$ to determine the phase shift needed for the basis functions in Eqs. 2 and 3, and the coefficients $V_{∥}$ and $V_{⊥}$ were found through numerical integration with $V_{m_{x}}$. For each trial, the background-subtracted coefficients were determined by subtracting the mean of the blank measurements immediately before and after the trial.

### Simultaneous imaging and inductive detection during linear fade

A linearly fading RMF was defined in Mathematica, exported, and loaded to the Keysight EDU33212A. A sample holder enabling visualization of the micromagnet when diffusely backlit from a vantage point above the setup (Fig. S2B) was attached at a fixed position at the approximate center of the inductively detectable volume. The setup was imaged from above at 60 frames per second using a Panasonic Lumix DMC-GX85 camera during simultaneous acquisition of the voltage signal. The inductive sensing data collection was repeated five times using the same micromagnet, detaching the sample arm to perform a blank measurement between each trial. Inductive signals were processed using a slightly modified version of the fixed frequency phase decomposition techniques described above, in which the limits of each full period of integration were taken to correspond to time indices at their center. To find phase, the arctangent of the ratio of the out-of-phase and in-phase components was taken. For image processing, the color video frames were cropped and converted to grayscale, and an intensity threshold was applied to produce binary images. MATLAB's built-in function “regionprops” was used to extract the orientation of the major axis of the best-fit ellipse for the white pixels corresponding to the micromagnet. This function returns orientation angles restricted to the range of −π/2 to π/2, so a custom function was used to correct discontinuities in the measured orientation of the micromagnet. Phase lag was found, up to a constant offset, by subtracting a linear angle versus time function reflecting the known time dependence of the field ϕ=ωt+C. The constant offset *C* for the optical phase lag was determined through a variational fitting algorithm minimizing the root mean squared error between the inductive phase angle and optical phase angle data.

### Signal processing for swept frequencies

Waveforms with the desired ω(t) characteristics were defined mathematically and loaded to the function generator, with an initial guess for the amplitude envelope versus time based on the known impedance of the RMF coils. The amplitude envelope versus time was then rescaled iteratively based on empirically observed $V_{H_{x}}$ and $V_{H_{y}}$ signals to produce a more constant magnitude. The same samples that were used for fixed frequency measurements were also used for swept frequency measurements, with five repeated trials interspersed with blanks. Internal oscilloscope averaging was set to eight for the slow sweeps. Analysis, in brief, consisted of fitting the $V_{H_{x}}$ curve with the known defined function to verify time delay and correct for minute time-scaling artifacts introduced by the oscilloscope (approximately ±2%). With appropriate time coordinate transformation, and with the signal normalized by the known instantaneous frequency, $V_{m_{x}}$ was used to determine $V_{∥}(t)$ and $V_{⊥}(t)\;$ through numerical integration as described in Eqs. 5 and 6. $V_{∥}(f)$ and $V_{⊥}(f)$ were found through the known dependence of f(t). Additional validation of this fitting technique can be found in the Supplementary material text and Fig. S3.

### Construction of the magic sphere

The structure designed to hold the magnet array depicted in Fig. 4A was 3D printed in four parts, with two of these quarters epoxied together to form two halves. Individual elements of permanent ferrite magnets consisted of stacked 20 mm × 20 mm × 3 mm blocks (grade Y35, Supermagnete.ch, FE-Q-20-20-03), forming approximate cubes of 20 mm side length, which were wrapped in electrical tape. The inner equatorial ring of magnets alternated between ferrite and neodymium iron boron with identical geometry (N45, Supermagnete.ch, Q-20-20-03-N). Magnets were loaded into their designated spaces, affixed by hot glue and epoxy. A total of 164 positions corresponded to ferrite magnet stacks, and 22 corresponded to interleaved ferrite and neodymium iron boron stacks, although the 28 magnet positions corresponding to the seam between the two hemispheres were left empty to supply a weaker gradient in the *y* direction while maintaining the symmetry needed to form a zero point. The two halves were brought together around the inductive detection coil using zip ties, with the zero point coinciding with the zone for inductive detection (see Fig. S4).

### Selection field experiments

A three-axis linear micropositioner (SmarAct, controller: HCU-3CX-USB-TAB, piezo positioner: SLC2445 series) was mounted atop the array of permanent magnets, and a 3D printed arm was used to hold either the three-axis Hall probe (Metrolab THM1176) or one of the several sample chambers (Fig. 4D; Fig. S7). Characterization of the SF and the superposition of the SF and RMF were performed with the Hall probe. Subsequently, the sample chamber was attached to the arm, and the zero point was located where the inductive signal from the micromagnet was maximized. Magnitude was varied at each frequency to empirically maximize out-of-phase signal. Inductive measurements were conducted along the *z* and *y* axes at known displacements relative to the zero point, as set by the micropositioner, with blank measurements taken before and after each sweep with the arm removed. In-phase and out-of-phase components were found through direct numerical integration of $V_{m_{x}}$ with normalized versions of $V_{H_{x}}$ and $V_{H_{y}}$ (with any constant offset subtracted and max amplitude set to 1). Blank values were subtracted, as determined from measurements conducted with the sample arm detached immediately prior to and after a sequence of measurements along an axis. For the experiments with visual feedback, a Panasonic Lumix DMC-GX85 camera was mounted above, and a diffuse light source was placed below the setup. For the resolution studies, resulting out-of-phase signal versus position curves were normalized to their maximum value or to the maximum value projected by a second-order spline interpolation in the case of rapidly varying curves.

## Supplementary Material

pgad297_Supplementary_Data

## Data Availability

All source data, analysis code, and STL files for the designs in this study have been made freely and permanently available through the ETH Zurich Repository for Publications and Research Data: https://doi.org/10.3929/ethz-b-000627109.
