# Supplementary material for: Inductive sensing of magnetic microrobots under actuation by rotating magnetic fields
Source: PNAS Nexus. 2023 Sep 12;2(9):pgad297. doi: 10.1093/pnasnexus/pgad297 (PMC10516638; doi:10.1093/pnasnexus/pgad297)
Supplement: pgad297_Supplementary_Data [file pgad297_supplementary_data.zip › PNASNEXUS-PNASNEXUS-2023-00424R-A-s04.pdf]

PNAS nexus

Supporting Information for

Inductive sensing of magnetic microrobots under actuation by rotating magnetic fields

Michael G. Christiansen*[1], Lucien Stöcklin[1,2], Cameron Forbrigger[1], Shashaank Abhinav Venkatesh [1,3], Simone Schuerle*[1]
1 Department of Health Sciences and Technology, ETH Zurich
2 Department of Biosystems Science and Engineering, ETH Zurich
3 Department of Biomedical Engineering, National University of Singapore
*Michael G. Christiansen and *Simone Schuerle
Email: michael.christiansen@hest.ethz.ch, simone.schuerle@hest.ethz.ch

This PDF file includes:

Supporting text
Figures S1 to S11
Description for Supplementary Movies S1 to S3
SI References

Supporting Information Text

Theoretical sensitivity analysis. Here, details are provided to substantiate the sensitivity analysis conducted for Fig. 2F of the main text. For simplicity, the geometry of the sense coils, which are partial cylindrical surfaces (Fig. 2A), are treated here approximately as partial spherical surfaces. Specifically, they are treated as latitude-longitude squares on the surface of a sphere of radius R with polar angle limits $\theta_1 \leq \theta \leq \theta_2$ and azimuth angle limits $\varphi_1 \leq \varphi \leq \varphi_2$ (Fig. S2).

Given a single loop of conducting wire that encloses a surface S, the voltage V induced in the loop by a magnetic field $\boldsymbol{B}$ is given by Faraday's law of electromagnetic induction:

$$V(t) = \frac{d}{dt}\left(\int_S \boldsymbol{B} \cdot \hat{\boldsymbol{n}}\, dA\right) \quad [1]$$

where t is time, dA is a differential area element of the surface S, and $\hat{\boldsymbol{n}}$ is the normal vector to that differential area element. A coil composed of N perfectly overlapping loops results in N times higher induced voltage.

Given a magnetic point dipole at a position $\boldsymbol{P}_m$, the magnetic field from the magnetic dipole can be determined analytically for any position of interest $\boldsymbol{P}$. The equation that describes the magnetic field from a magnetic point dipole in 3D space as a function of the relative position $\boldsymbol{r} = \boldsymbol{P} - \boldsymbol{P}_m$ is:

$$\boldsymbol{B}_m = \frac{\mu_0 m}{4\pi\|\boldsymbol{r}\|^3}(3\hat{\boldsymbol{r}}\hat{\boldsymbol{r}}^{\mathrm{T}} - I_3)\hat{\boldsymbol{m}} \quad [2]$$

where μ_0 is the permeability of free space, $\|\boldsymbol{r}\|$ is the 2-norm of $\boldsymbol{r}$, $\hat{\boldsymbol{r}} = \boldsymbol{r}/\|\boldsymbol{r}\|$ is a unit vector in the direction of r, and I_3 is a 3 × 3 identity matrix (1).

For a dipole rotating within the xy plane at a constant angular velocity ω, the resulting magnetic field expressed in spherical coordinates becomes

$$\boldsymbol{B}_m(t) = \frac{\mu_0 m}{4\pi r^3}\begin{bmatrix} 2\sin\theta\cos(\omega t - \varphi) \\ -\cos\theta\cos(\omega t - \varphi) \\ -\sin(\omega t - \varphi) \end{bmatrix} \quad [3]$$

Substituting Eq. 3 into Eq. 1 in order find an estimate for the induced voltage contribution of the dipole V_m,

$$V_m(t) = \frac{\mu_0 m}{2\pi R}\frac{d}{dt}\left(\int_{\varphi_1}^{\varphi_2}\int_{\theta_1}^{\theta_2}\cos(\omega t - \varphi)\sin^2\theta\, d\theta d\varphi\right) \quad [4]$$

For this simplified case, the solution is analytically tractable, and can be expressed in terms of the following parameters:

$$\theta_\Delta = \frac{\theta_2 - \theta_1}{2} \quad [5]$$

$$\bar{\theta} = \frac{\theta_1 + \theta_2}{2} \quad [6]$$

$$A_\theta = 2\theta_\Delta - \sin(2\theta_\Delta)\cos(2\bar{\theta}) \quad [7]$$

$$\varphi_\Delta = \frac{\varphi_2 - \varphi_1}{2} \quad [8]$$

$$\bar{\varphi} = \frac{\varphi_1 + \varphi_2}{2} \quad [9]$$

$$A_\varphi = \sin(\varphi_\Delta) \quad [10]$$

The full expression for $V_m(t)$ can then be given as

$$V_m(t) = -\frac{\mu_0 m\omega}{2\pi R} A_\theta A_\varphi \sin(\omega t - \bar{\varphi}) \quad [11]$$

The parameters A_θ and A_φ are unitless geometric factors; therefore we can see that the voltage induced by a rotating dipole scales with $1/L$, where L is the characteristic length of the coils. As expected, the induced voltage scales linearly with respect to the dipole magnitude m and the rotation frequency ω. The average azimuth angle $\bar{\varphi}$ of the coil only results in a phase shift of the induced voltage signal and has no effect on the magnitude of the signal. The effect of φ_Δ is also relatively simple: A_φ is maximized at $\varphi_\Delta = \pi/2$, $A_\varphi = 1$ (when the width of the coil spans a full 180°) and decreases symmetrically about that maximum.

The method of physical background subtraction shown in Fig. 2A, in which the voltage induced by the rotating magnetic moment is ultimately taken from the wiper of the potentiometer at the center of a voltage divider, results in the magnitude of the sensed voltage dropping by a factor of two. The voltage that is sensed by the oscilloscope is also amplified, increasing the scale of the measured voltage by a factor of K_g, where K_g is the linear gain of the amplifier. By grouping the relevant parameters determined by the coil setup (coil parameter) and constrained by the microrobot (agent parameter) the expected scale of measured voltages can be mapped over many orders of magnitude (main text, Fig. 2F).

It is enlightening to further consider the scale of the voltages induced in the sense coils by the RMF itself. This enables both a comparison between the magnitude of the voltage induced by the RMF and the microrobot (“signal to background ratio” SBR) as well as the extent of background suppression that was ultimately achieved with our setup (“background rejection ratio” BRR). In spherical coordinates, a field with the same plane of rotation as the one assumed for m and also rotating at constant angular velocity ω can be expressed as follows:

$$\boldsymbol{B}_{RMF}(t) = B_{RMF} \begin{bmatrix} \sin\theta\cos(\omega t - \varphi) \\ \cos\theta\cos(\omega t - \varphi) \\ \sin(\omega t - \varphi) \end{bmatrix} \quad [12]$$

Substituting this expression into Eq. 1 yields

$$V_{RMF}(t) = -B_{RMF} R^2 \omega A_\theta A_\varphi \sin(\omega t - \bar{\varphi}) \quad [13]$$

Comparing Eq. 11 and 13, the time dependent part of this function and the geometric factor are identical and will drop out of a ratio, although it should be noted that a real microrobot would experience a phase lag between field and the moment for reasons discussed in the main text. Dividing Eq. 11 by Eq. 13 gives the SBR,

$$SBR = \frac{\mu_0 m}{2\pi B R^3} \quad [14]$$

For the microrobots used in this study, m is approximately 4.12×10^{-5} Am^{-2}, R was approximately 19.5 mm, and the SBR evaluates to values on the order of 10^{-4} to 10^{-3} for field magnitudes in the range of 1 to 10 mT.

The *BRR* for the representative traces in Fig. 2C can also be estimated. The residual background at 3.98 Hz and 7.2 mT observed for the blank sample can be estimated through fitting to have an amplitude of 9.725 mV. At this frequency, the characterized gain of the amplifier K_g was about 3255, implying the actual residual voltage was about 2.988 μV. For the geometry and turn number of turns present in the sense coil, $\theta_\Delta \approx \varphi_\Delta \approx \pi/4$, $N = 5000$, and $R \approx 19.5\ \mathrm{mm}$, giving an estimated voltage within the sense coil from the stated RMF of 138.2 mV. This suggests a BRR value of 4.62 × 10^4, or 93.3 dB. Since the residual signal and the uncompensated voltages occurring in the sense coils should scale similarly with B_{RMF} and ω, the BRR should remain about the same for all conditions investigated, while the apparent magnitude of the residual voltage increases. This analysis suggests that, after background had been properly adjusted, a background suppression of about 90 dB could be achieved with this setup.

Validation of frequency sweep phase decomposition analysis. Some additional details can be provided regarding phase decomposition with the frequency sweeps in the main text. For the case of a linear sweep with a constant rate α,

$$\omega(t) = \alpha t \quad [15]$$

Here, the weighting function $W(t)$ is as follows

$$W(t) = \frac{d}{dt}[\omega(t)t] = \frac{d}{dt}(\alpha t^2) = 2\alpha t \quad [16]$$

Advancing a single period after an initial time t_i to a final time t_f, it is possible to find an expression for t_f in terms of t_i and α

$$\alpha {t_f}^2 = \alpha {t_i}^2 + 2\pi \quad [17]$$

$$t_f = \sqrt{{t_i}^2 + \frac{2\pi}{\alpha}} \quad [18]$$

The angular frequency corresponding to these limits can be taken as the time average $\langle\omega(t)\rangle$ over a single period. Here,

$$\langle\omega(t)\rangle = \frac{1}{t_f - t_i}\int_{t_i}^{t_f}\omega(t) = \frac{1}{2}\frac{\alpha\left({t_f}^2 - {t_i}^2\right)}{2\pi\left(t_f - t_i\right)} = \frac{\alpha(t_f - t_i)(t_f + t_i)}{4\pi(t_f - t_i)} = \frac{\alpha(t_f + t_i)}{4\pi} \quad [19]$$

In the case of the linear sweep results shown in the main text, $\alpha = 400\pi$. To ensure a smooth rise and fall of the field, sigmoidal step functions are introduced such that the full expression for the desired field as a function of time is as follows:

$$H_x(t) = H_0\cos(400\pi t^2)\left\{\frac{1}{1 + \exp[-150(t - 0.025)]} - \frac{1}{1 + \exp[-150(t - 0.575)]}\right\} \quad [20]$$

$$H_y(t) = H_0\sin(400\pi t^2)\left\{\frac{1}{1 + \exp[-150(t - 0.025)]} - \frac{1}{1 + \exp[-150(t - 0.575)]}\right\} \quad [21]$$

These functions, as well as their modulus, are plotted in Fig. S3A. The time of constant field magnitude is approximately [0.05,0.55]. A more important constraint for performing phase decomposition with the approximate forms in Eq. 5 and 6 from the main text is to verify orthogonality of the basis functions. In other words, for Eq. 5 and 6 to describe a valid approximation, the following should be true for each set of suitable limits:

$$\frac{2\int_{t_i}^{t_f} W(t)\cos^2[\omega(t)t]\,dt}{\omega(t_f)t_f - \omega(t_i)t_i} \approx \frac{2\int_{t_i}^{t_f} W(t)[\mathrm{H_x}(\mathrm{t})/H_0]^2 dt}{\omega(t_f)t_f - \omega(t_i)t_i} \approx 1 \quad [22]$$

$$\frac{2\int_{t_i}^{t_f} W(t)\sin^2[\omega(t)t]\,dt}{\omega(t_f)t_f - \omega(t_i)t_i} \approx \frac{2\int_{t_i}^{t_f} W(t)\left[\mathrm{H_y}(\mathrm{t})/H_0\right]^2 dt}{\omega(t_f)t_f - \omega(t_i)t_i} \approx 1 \quad [23]$$

$$\frac{2\int_{t_i}^{t_f} W(t)\sin[\omega(t)t]\cos[\omega(t)t]\,dt}{\omega(t_f)t_f - \omega(t_i)t_i} \approx \frac{2\int_{t_i}^{t_f} W(t)\left[\mathrm{H_x(t)H}_y\mathrm{(t)}/{H_0}^2\right]dt}{\omega(t_f)t_f - \omega(t_i)t_i} \approx 0 \qquad [24]$$

For the case of the linear sweep, good convergence to these expected values is observed (Fig S3B). The absolute value of the error function is also plotted to quantify deviation from orthogonality (Fig. S3C). All points where the error falls below 0.01 were taken to be valid and included later in the analysis of actual signals. At this stage the deviation is purely numerical—this does not include the influence of noise or instrumental error in actual signals. The symmetry of the error function suggests that, in this case, the main source of error may be the residual influence of the sigmoidal step functions.

This process can also be repeated explicitly for a quadratic sweep with prefactor β:

$$\omega(t) = \beta t^2 \qquad [25]$$

In this case,

$$W(t) = \frac{d}{dt}[\omega(t)t] = \frac{d}{dt}(\beta t^3) = 3\beta t^2 \qquad [26]$$

The expression relating the limits t_i and t_f reduces to:

$$\beta {t_f}^3 = \beta {t_i}^3 + 2\pi \qquad [27]$$

$$t_f = \left({t_i}^3 + \frac{2\pi}{\beta}\right)^{1/3} \qquad [28]$$

Once again, the angular frequency these limits correspond to can be found using the time averaged value of $\omega(t)$:

$$\begin{aligned}\langle\omega(t)\rangle &= \frac{1}{t_f - t_i}\int_{t_i}^{t_f}\frac{\omega(t)}{2\pi} = \frac{1}{6\pi}\frac{\beta\left({t_f}^3 - {t_i}^3\right)}{t_f - t_i} = \frac{\beta(t_f - t_i)\left({t_f}^2 + t_f t_i + {t_i}^2\right)}{6\pi(t_f - t_i)} \\ &= \frac{\beta}{6\pi}\left({t_f}^2 + t_f t_i + {t_i}^2\right)\end{aligned} \qquad [29]$$

In the case of the quadratic sweep in the main text, $\beta = 2\pi$ and the time of constant field is from approximately [0.5,5.5]. The explicit forms of $H_x(t)$ and $H_y(t)$ are as follows:

$$H_x(t) = H_0\cos(2\pi t^3)\left\{\frac{1}{1+\exp[-15(t-0.5)]} - \frac{1}{1+\exp[-15(t-5.5)]}\right\} \qquad [30]$$

$$H_y(t) = H_0\sin(2\pi t^3)\left\{\frac{1}{1+\exp[-15(t-0.5)]} - \frac{1}{1+\exp[-15(t-5.5)]}\right\} \qquad [31]$$

These are shown in Fig. S3D. The same check on orthogonality described in Eq. 22-24 can be performed (Fig. S3E and Fig S3F). Here, the errors are larger than in the linear case, but convergence falls below an error of 0.01 for points that were considered in the analysis.

Supporting details for comparison to alternative magnetic sensors. Assumptions and additional information are provided here to substantiate claims made in the conclusion relating to the comparison of these inductive methods to other methods of sensing stray fields from one or more microrobots.

Supposing that Hall sensors or other similar sensors that essentially make field measurements at a single point in space were employed, they would need to detect the contribution from the stray field from the micromagnet (approximately 1 μT), which is about 10^4 times smaller than the RMF (on the order of 10 mT) when placed at the distance of the coils. This quantity can be estimated using Eq. S13 as follows

$$|\boldsymbol{B}_m| \leq \frac{\mu_0 m}{2\pi\|\boldsymbol{r}\|^3} = \frac{(4\pi \times 10^{-7}\mathrm{N\,A^{-2}})(4.12 \times 10^{-5}\mathrm{A\,m^{-2}})}{2\pi\,(0.0195\ \mathrm{m})^3} \approx 1.1\ \mu\mathrm{T} \qquad [32]$$

This implies that Hall sensors suitable to produce similar data to e.g. Fig. 3 of the main text during simultaneous RMF actuation would need to be able to sense combined fields of 10s of mT with at least ±0.01% precision at sample rates exceeding 10s or 100s of Hz. These are demanding technical specifications, and sensors that begin to approach them are likely to be expensive. For instance, the Lakeshore Model 475 Gauss probe is advertised as being capable of resolving 0.1 μT (10% of the expected signal magnitude from the microrobot) when measuring DC fields, within a range of 35 mT. It is unclear whether these specifications require filtering or averaging, but assuming they could measure with sufficient temporal resolution, this device might be sufficient.

By contrast, the techniques we demonstrated in our manuscript used simple geometric symmetry, cancellation techniques, and inexpensive signal amplifiers for inductive sensing. Because the measurements are at low frequency, even relatively basic oscilloscopes could enable the measurement of multiple channels. (An end stage product or device would likely use some alternative to the oscilloscope to reduce cost.) Moreover, we are optimistic that we can improve the sensitivity of our setups, whereas the example of the Gauss probe above is already a highly optimized scientific instrument.

As mentioned in the main text, another major difference is in the scaling behavior. We showed that by leaving the properties of the microrobot constant, and uniformly scaling up the entire sense coils, the signal drops as 1/R. The scaling situation is substantially worse for point measurements with e.g. Hall probes, which would simply drop off as $1/R^3$ as the distance between the source increases, based on Eq. S13.

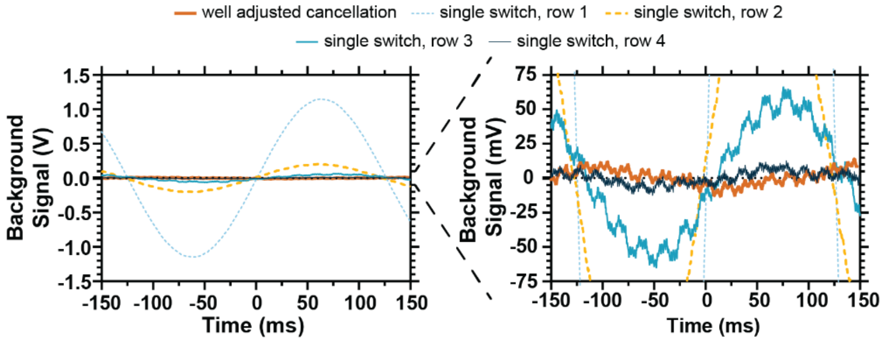

Switch level	Adjustment magnitude of single switch (mV/Hz/mT)
Row 1	40.
Row 2	7.2 (×5.6)
Row 3	2.1 (×3.4)
Row 4	0.35 (×6.0)
differences between loops in Row 4	<0.3

Fig. S1. Characterization of performance of the phase adjustment loops. As described in the main text, the out-of-phase adjustment device consisted of a switch box with 4 rows of 5 switches each that could selectively incorporate phase correcting loops into the sense and compensation coil. Here, inductive signals are shown for a blank sample exposed to an RMF at 3.98 Hz and 7.2 mT. The system was first well adjusted for background cancellation and an example switch from each row was flipped to examine the residual signal that was reintroduced. "Row 1" corresponds to the phase correcting loops with the highest area, and they decrease progressively in area until "Row 4", which is comprised of the smallest loops. Both plots show the same data, with the y axis rescaled in the second. A table is provided with estimates of the approximate adjustment magnitude of single switches in each row. The factor of decrease in adjustment magnitude between each row is shown in grey. Note that users could also choose between different switches in each row, which differ minutely in their adjustment factor. This effectively provided another level of adjustment.

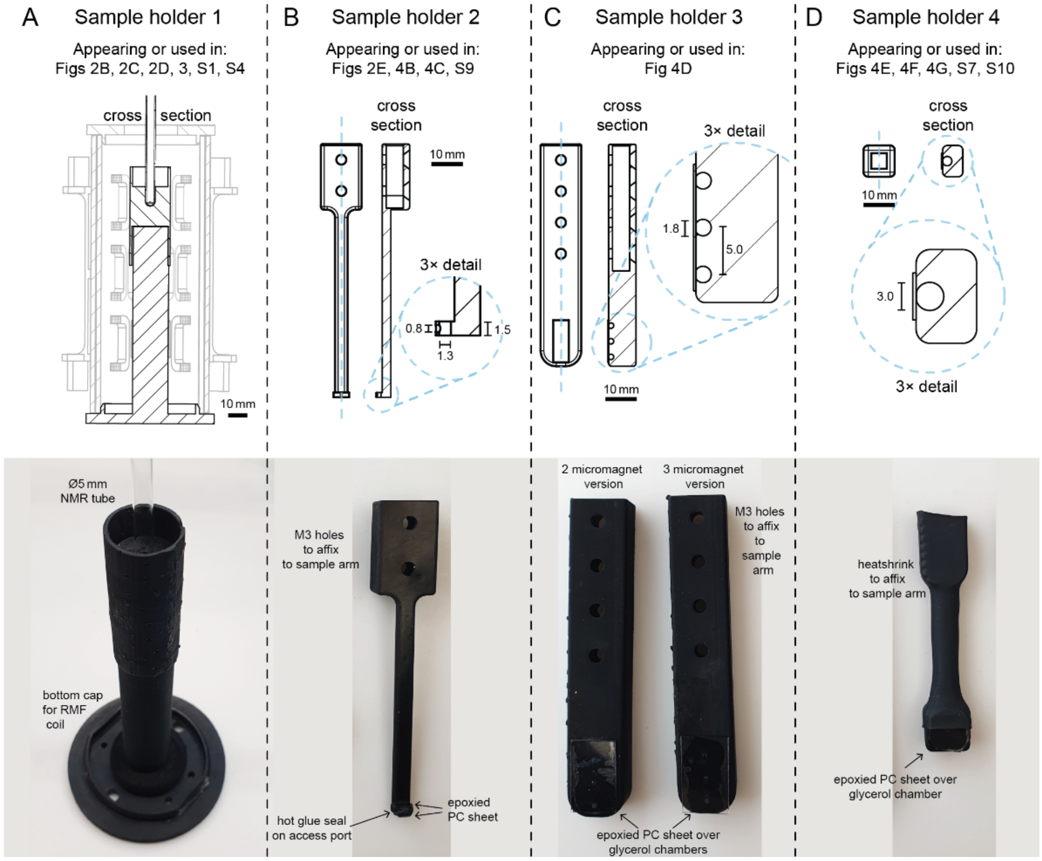

Fig. S2. Summary of sample holders used in various experiments. For all sample holder types shown, the basic design, specific occurrences of their use in this study, and photographs are shown. Unlabeled dimensions are given in mm. All holders were 3D printed. (*A*) This sample holder was intended for basic measurements at a position fixed in the center of the inductive sensing region. A modified version of the end cap aligning the RMF coil with the sense/compensation coil apparatus featured a solid pillar fitted with a cap designed to accept a 5 mm NMR tube. (*B*) This sample holder was specially designed to enable visualization of the micromagnet from above and was made with a relatively narrow arm supporting the sample chamber to avoid restricting the range of motion of the holder. Polycarbonate (PC) sheets were carefully epoxied on either side of the main Ø1.3 mm cylindrical chamber. A Ø0.8 mm access port for filling glycerol, degassing to remove bubbles, and adding the micromagnet was provided in the front and sealed with hot glue after loading. (*C*) This holder was specially designed for the mutli-magnet actuation experiment and features several Ø1.8 spherical cavities spaced vertically at 5.0 mm intervals, filled with glycerol, and (potentially) loaded with a micromagnet. A PC sheet was epoxied to the front to seal the chambers after loading their contents. (*D*) This sample holder was employed for the spatial selectivity measurements and features a single Ø3.0 mm spherical cavity loaded and sealed in a manner similar to the previous sample holder. In this case, heat shrink made rigid after heating was used to affix the sample holder to the polymer arm connecting it to the micropositioner.

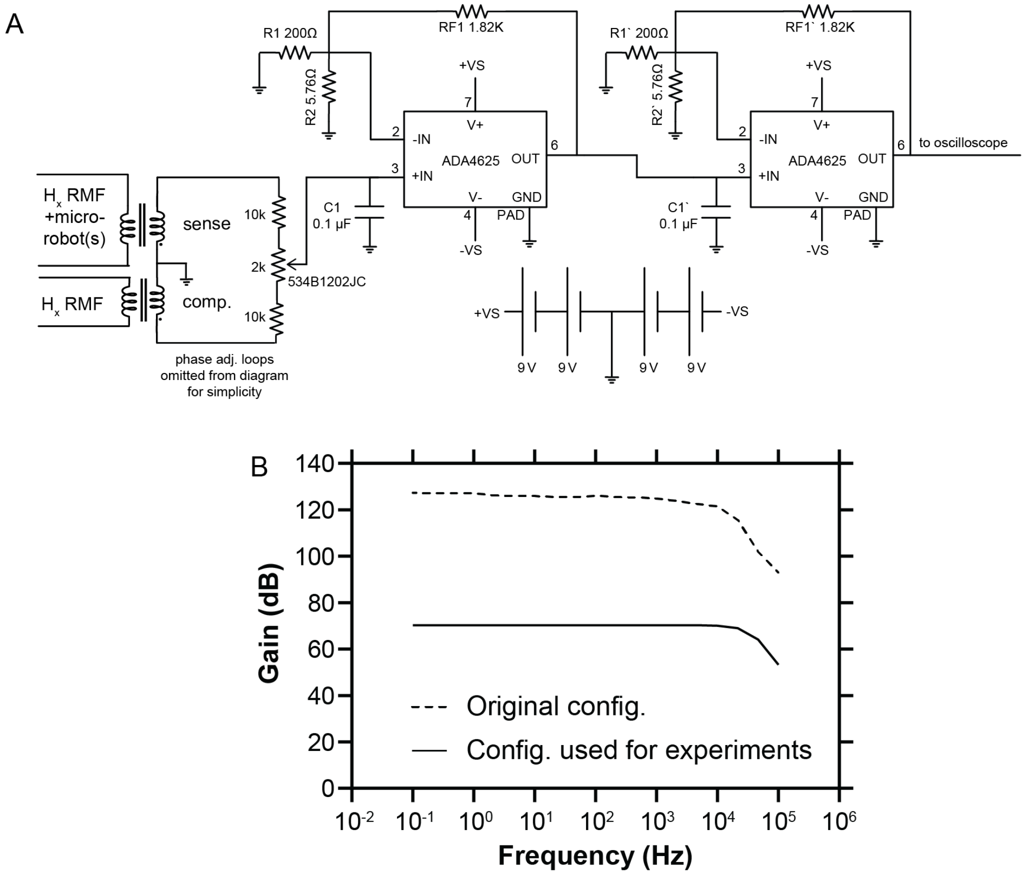

Fig. S3. Additional information about signal amplification in the experiments described in the main text. (*A*) A more detailed schematic of the amplifier circuit is shown. For PCB layout, two of the evaluation boards EVAL-ADA4625-1ARDZ were used with the component values indicated. C1 was found to be necessary to suppress self-resonance of the detection coils. Gain was set by changing RF1 and R2, or RF1' and R2'. The signal amplifier was powered by standard 9 V batteries to fully avoid the possibility for noise from a rectified DC supply. (*B*) Measured gain versus frequency is shown for an initial configuration of the amplifier, which achieved a gain of more than 120 dB, as well as the gain setting used for experiments (70 dB). The gain was reduced so that the micromagnet could be observed at the highest frequencies. It was also beneficial to reduce the gain because additional suppression of the ambient 50 Hz noise is needed to avoid clipping output waveforms.

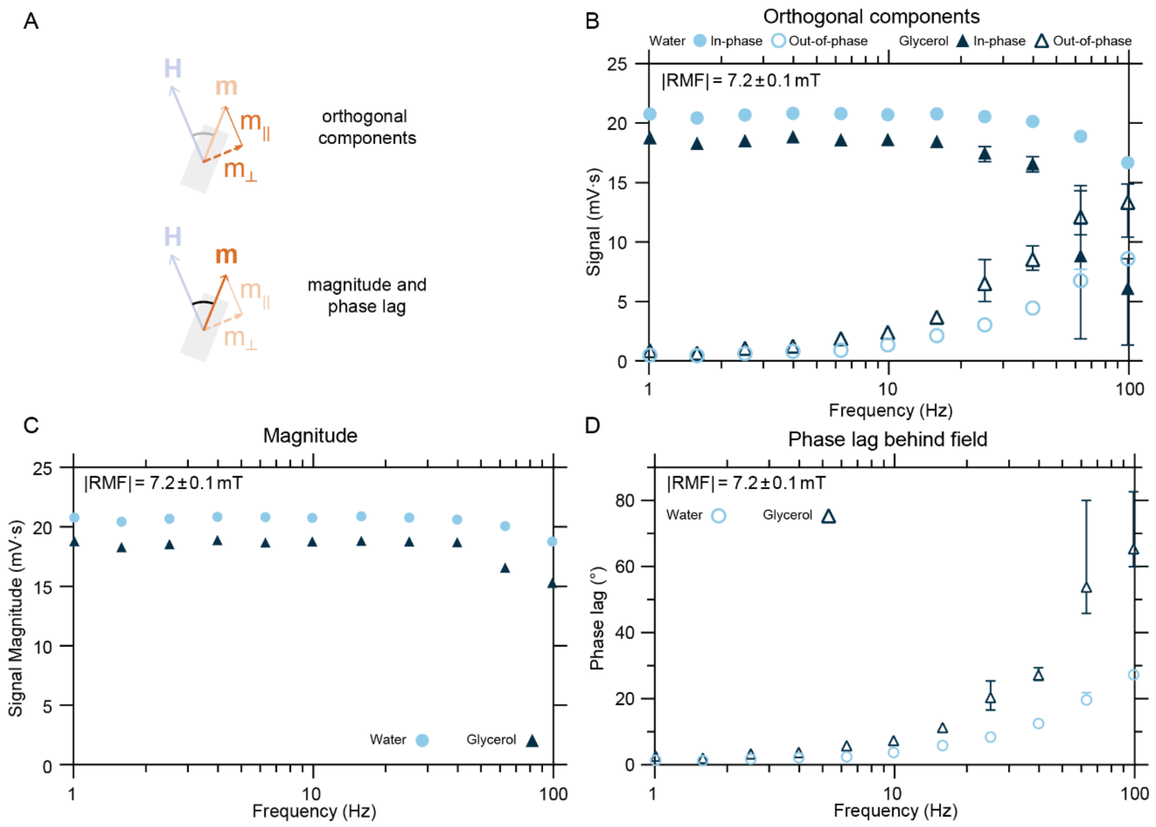

Fig. S4. Discreet frequency sweep for micromagnets in glycerol or water. (*A*) A sketch based on Fig. *1* of the main text emphasizes how the same inductive signal from a micromagnet can be equivalently represented with orthogonal components or with magnitude and phase. (*B*) Signal collection and phase decomposition was performed over a logarithmic sweep of fixed frequencies for a micromagnet in water and a micromagnet in glycerol. Data points represent the mean of 5 technical replicates with the sample unloaded and a blank sample measured between each replicate. Error bars represent the 95% confidence interval. The same results are represented in terms of (*C*) magnitude and (*D*) phase lag.

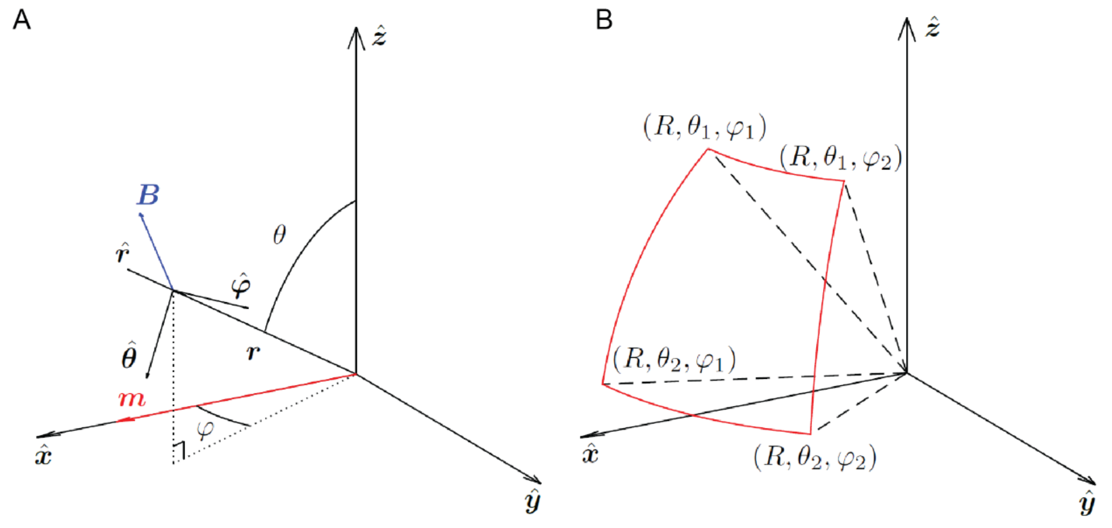

Fig. S5. Explanatory sketch of geometry for theoretical sensitivity analysis. (*A*) The magnetic field vector $\boldsymbol{B}$ at a position $\boldsymbol{r}$ relative to a point dipole with dipole moment vector $\boldsymbol{m}$. Both Cartesian $\hat{\boldsymbol{x}}\hat{\boldsymbol{y}}\hat{\boldsymbol{z}}$ and spherical $\hat{\boldsymbol{r}}\hat{\boldsymbol{\theta}}\hat{\boldsymbol{\varphi}}$ coordinate frames are shown. The Cartesian frame and the spherical frame have the same origin, but the spherical frame vectors are drawn at the tip of $\boldsymbol{r}$ to allow for clearer visualization. (*B*) A loop of wire that forms a latitude-longitude rectangle $\boldsymbol{\theta_1} \leq \boldsymbol{\theta} \leq \boldsymbol{\theta_2}$, $\boldsymbol{\varphi_1} \leq \boldsymbol{\varphi} \leq \boldsymbol{\varphi_2}$ on the surface of a sphere with radius $\boldsymbol{R}$ centered at the origin. Note that the coils actually used in the apparatus described in the main text were partial cylindrical surfaces, which here have been approximated in terms of latitude longitude squares to simplify the analytical problem.

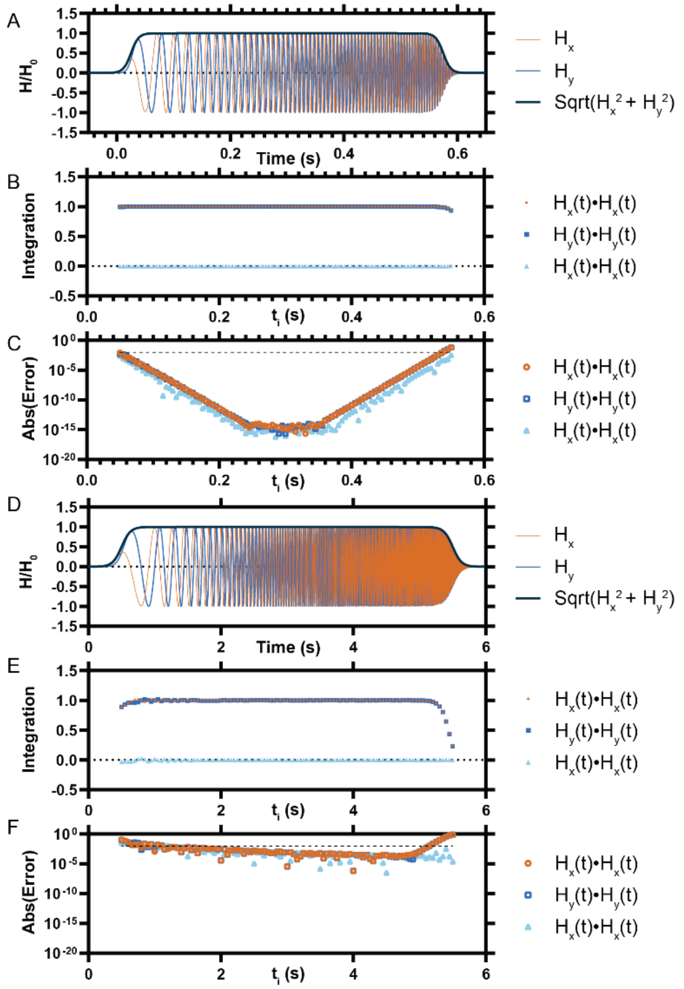

Fig. S6. Numerical validation of phase decomposition with swept frequencies for the cases shown in the main text. Panels (*A*)-(*C*) pertain to the linear sweep from approximately 10 to 100 Hz described in Fig. 3A of the main text. (*A*) The desired field as a function of time, normalized to the RMF magnitude $\boldsymbol{H_0}$ is shown in terms of its components $\boldsymbol{H_x}$ and $\boldsymbol{H_y}$. (*B*) The orthogonality of these functions when integrated with a weighting function and appropriately defined limits is tested by assessing convergence to 1 or 0. As simplified notation, $\boldsymbol{H_x(t)\cdot H_x(t)}$ is used to denote results from Eq. 22, $\boldsymbol{H_y(t)\cdot H_y(t)}$ for Eq. 23, and $\boldsymbol{H_x(t)\cdot H_y(t)}$ for Eq. 24. (*C*) To quantify the level of convergence, the absolute value of the difference between calculated and expected values is shown. Panels (*D*)-(*F*) pertain to the quadratic sweep from approximately 0.6 Hz to 26 Hz described in Fig. 3B of the main text and are directly analogous to (*A*)-(*C*). It should be noted in (*F*) that the error is higher in the case of the quadratic sweep, but still acceptably low for the timeframe of interest.

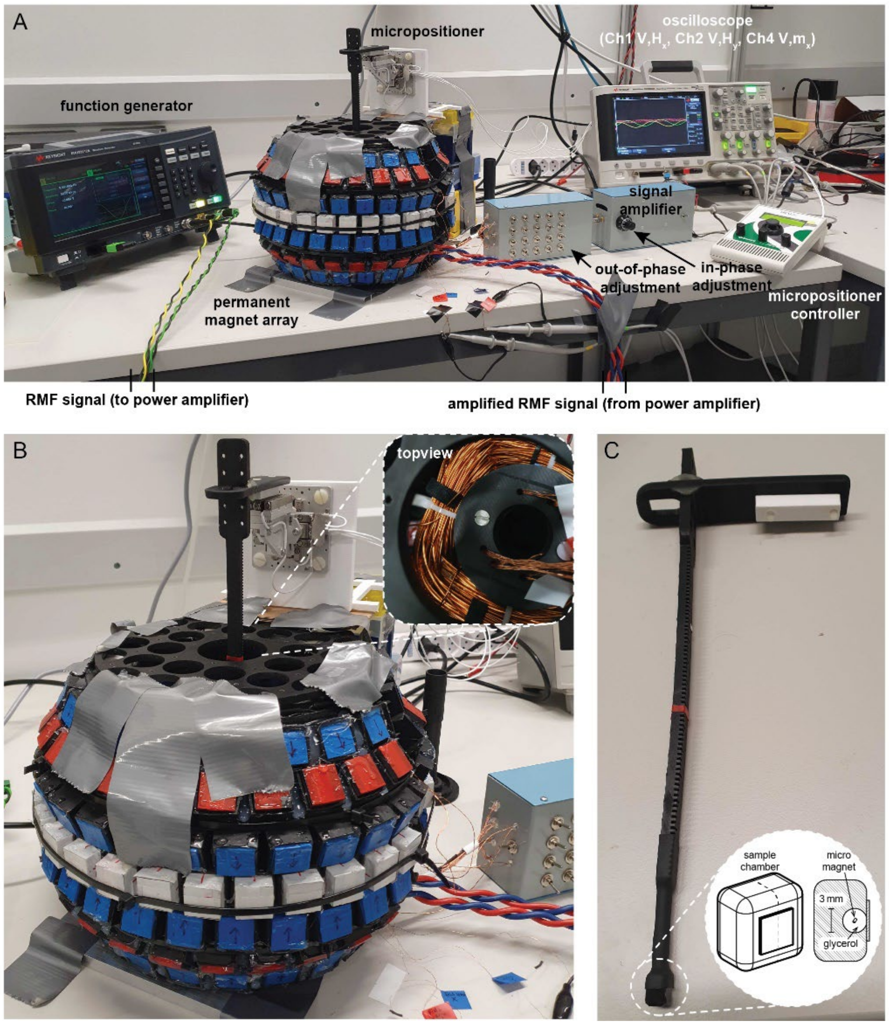

Fig. S7. Images of the selection field setup. (*A*) The full setup is depicted as actually used to collect the data appearing in Fig. 4 of the main text, with the components labelled. The power amplifier is not included because it was relocated across the room to reduce 50 Hz noise, but its inputs and outputs are labelled. (*B*) A detailed view of the permanent magnet array used to generate the zero point, along with the micro positioner mounted atop it. A topview is provided with the arm holding the sample removed (inset). (*C*) The arm that holds the sample chamber and attaches to the micropositioner stage is shown. The sample chamber is affixed to the arm using a piece of heat shrink.

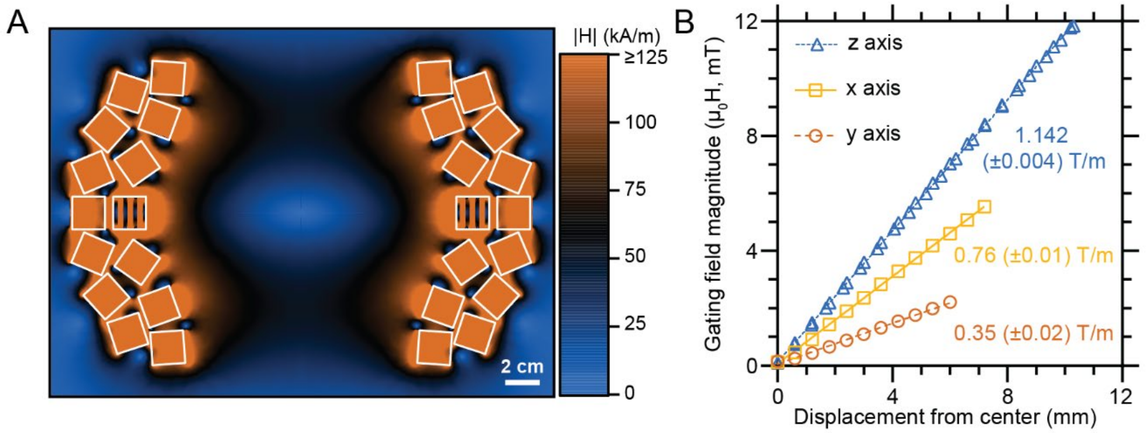

Fig. S8. Simulation and characterization of the "magic sphere" permanent magnet array. (*A*) Cross sectional representation of a finite element simulation of the GF produced by an azimuthally symmetric approximation of the permanent magnet array. Outlines of stacks of magnets are overlaid in white. Made with FEMM (2). See Fig 4A of the main text for additional context. (B) Magnetostatic field magnitude measured by Hall probe as a function of displacement from the zero point along each of the indicated axes. Slopes extracted from separate linear regressions are shown with 95% confidence interval bounds.

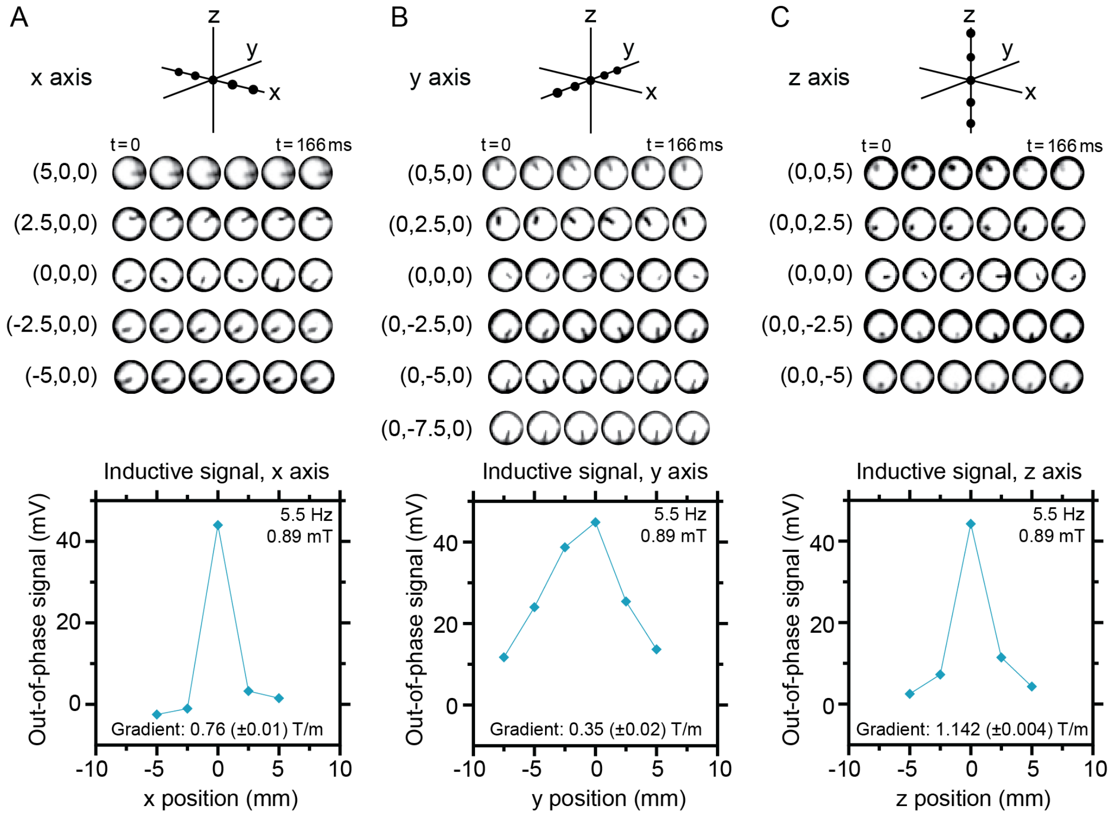

Fig. S9. Simultaneous visualization and inductive detection in a magnetostatic gating field. For each axis of points, still frames from videos taken over approximately one period of rotation of the RMF are shown for a single micromagnet moved to various indicated positions. The sample chamber has a diameter of 1.3 mm. The out-of-phase component of the simultaneous inductive measurement is shown below. (*A*) corresponds to a set of points on the x axis, (*B*) corresponds to points on the y axis, and (*C*) corresponds to points on the z axis. Note that, due to glare preventing visualization of the main chamber, the image representing (5,0,0) is reconstructed from the specular reflection of the chamber. See main Fig. 4B for a photograph of the chamber in the selection field setup. Please also see Supplementary Movies 2 and 3.

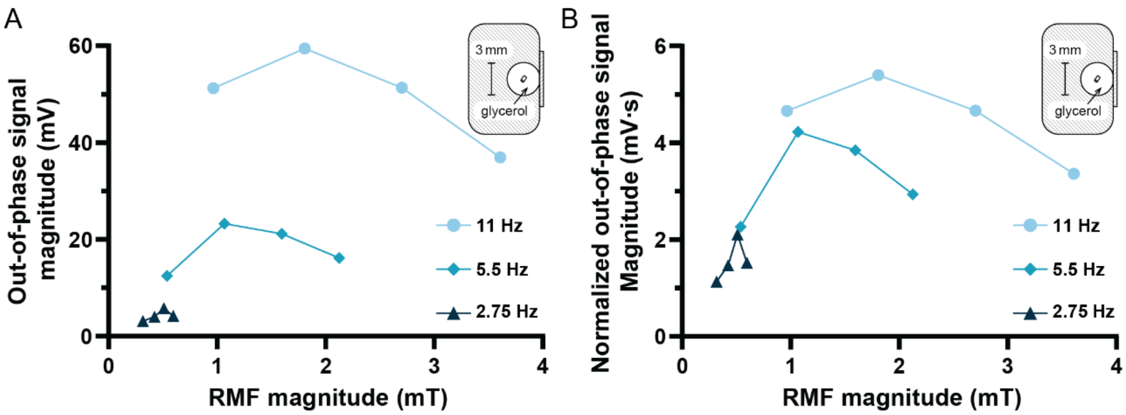

Fig. S10. Empirical determination of RMF conditions suitable for the selection field experiments. (*A*) Out-of-phase signal amplitudes are shown as a function of RMF magnitude for the sample chamber placed as close to the zero point as possible. As expected, the out-of-phase signal increases with RMF magnitude, but then drops again as the signal from the magnet is dominated by the in-phase component. As discussed in the main text, these signals are proportional to the rate of irreversible work done on the micromagnet, so the peak corresponds roughly to the step out frequency. (*B*) By normalizing the same data to frequency, it can be replotted in terms of quantities that reflect the magnitude of the irreversible torque or the irreversible work per cycle of the field.

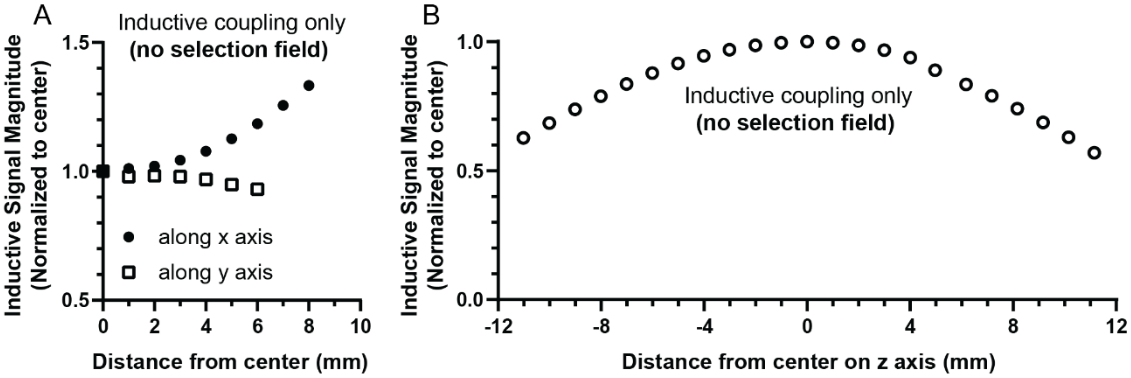

Fig. S11. Assessment of spatial variation of inductive coupling in absence of selection field. To ensure a large signal at low frequency, a cylindrical magnet of diameter 750 µm and length of 1000 µm (N50, SM Magnetics Cyl0010-25) was placed in glycerol in a 5 mm NMR tube and its position was varied by the microcontroller under constant RMF. Variation in magnitude of the induced signal under these conditions was assumed to arise from spatial variation of inductive coupling to the sense coils. (*A*) Variation in signal magnitude along the x and y axes is shown, normalized to the center. Notably, the observed inductive coupling is more constant for variation in the y direction than the x direction for this setup. (*B*) Variation of the inductive signal magnitude is shown in the z direction. To facilitate comparison, the ordinate axes of these plots have been kept to scale with each other. Variation of inductive coupling in the z direction is clearly much weaker than the influence of the selection field.

Movie S1 (separate file). Video at 0.25 actual speed of cylindrical micromagnet (300 μm diameter, 500 μm length) exposed to a linearly decaying rotating magnetic field with a frequency of 5.5 Hz. Inset shows simultaneous zoom of sample chamber. Video tracks two full linear fade events. This video data was processed to determine optically extracted phase lag in Fig. 2E.

Movie S2 (separate file). Video at 0.25 actual speed showing the response of the same micromagnet to a combined rotating magnetic field and magnetostatic gradient field at various points along the z axis. At the zero point, unsuppressed rotation occurs. Above and below the zero point, the magnet precesses, with increasingly limited motion as the gating field becomes larger at greater distance from the zero point. Note that these trials were conducted sequentially with the same micromagnet and that they are arrayed along a line to facilitate mutual comparison. See also Figs. 4B and S9C for additional context.

Movie S3 (separate file). Video at 0.25 actual speed showing the response of the same micromagnet to a combined rotating magnetic field and magnetostatic gradient field at various points along the x and y axes. At the zero point, unsuppressed rotation occurs. Away from the zero point, the magnet “wiggles”, with increasingly limited motion as the gating field becomes larger at greater distance from the zero point. Note that these trials were conducted sequentially with the same micromagnet and that they are arrayed along the axes to facilitate visual comparison. See also Figs. 4B, S9A, and S9B for additional context.

SI References

1. J. J. Abbott, E. Diller, A. J. Petruska, Magnetic Methods in Robotics. *Annual Review of Control, Robotics, and Autonomous Systems* **3**, 57-90 (2020).
2. D. Meeker, Finite Element Method Magnetics Version 4.2. https://www.femm.info/wiki/Documentation/ (2020)
